# Functional Genomics Identifies Tis21-Dependent Mechanisms and Putative Cancer Drug Targets Underlying Medulloblastoma Shh-Type Development

**DOI:** 10.3389/fphar.2016.00449

**Published:** 2016-11-30

**Authors:** Giulia Gentile, Manuela Ceccarelli, Laura Micheli, Felice Tirone, Sebastiano Cavallaro

**Affiliations:** ^1^Institute of Neurological Sciences, National Research CouncilCatania, Italy; ^2^Institute of Cell Biology and Neurobiology, National Research Council, Fondazione Santa LuciaRome, Italy

**Keywords:** medulloblastoma model, cerebellar precursor cell, drug target, Sonic Hedgehog, primary cilium, neural migration, retina, chemokines

## Abstract

We have recently generated a novel medulloblastoma (MB) mouse model with activation of the Shh pathway and lacking the MB suppressor Tis21 (*Patched1*^+/−^*/Tis21*^*KO*^). Its main phenotype is a defect of migration of the cerebellar granule precursor cells (GCPs). By genomic analysis of GCPs *in vivo*, we identified as drug target and major responsible of this defect the down-regulation of the promigratory chemokine Cxcl3. Consequently, the GCPs remain longer in the cerebellum proliferative area, and the MB frequency is enhanced. Here, we further analyzed the genes deregulated in a *Tis21*-dependent manner (*Patched1*^+/−^*/Tis21* wild-type vs. *Ptch1*^+/−^*/Tis21* knockout), among which are a number of down-regulated tumor inhibitors and up-regulated tumor facilitators, focusing on pathways potentially involved in the tumorigenesis and on putative new drug targets. The data analysis using bioinformatic tools revealed: (i) a link between the Shh signaling and the *Tis21*-dependent impairment of the GCPs migration, through a Shh-dependent deregulation of the clathrin-mediated chemotaxis operating in the primary cilium through the Cxcl3-Cxcr2 axis; (ii) a possible lineage shift of Shh-type GCPs toward retinal precursor phenotype, i.e., the neural cell type involved in group 3 MB; (iii) the identification of a subset of putative drug targets for MB, involved, among the others, in the regulation of Hippo signaling and centrosome assembly. Finally, our findings define also the role of Tis21 in the regulation of gene expression, through epigenetic and RNA processing mechanisms, influencing the fate of the GCPs.

## Introduction

About 30% of medulloblastomas (MBs), the pediatric tumor of the cerebellum, originates from the granule neuron precursor cells (GCPs) located in the external granular layer (EGL), at the surface of the developing cerebellum, in consequence of hyperactivation of the Sonic Hedgehog (Shh) pathway (Kadin et al., 1970; Schüller et al., 2008; Yang et al., 2008; Gibson et al., 2010; Northcott et al., 2012). Other MB subtypes may originate from neural precursors of the cerebellar embryonic anlage, different from GCPs and dependent on Wnt signaling, or from GCPs with activation of different pathways (group 3), or also from neural precursors of unknown origin (group 4; Northcott et al., 2012). GCPs intensely proliferate postnatally in the EGL, before exiting the cell cycle and migrating inward to form the mature internal granular layer (IGL; Hatten, 1999). GCPs in the EGL are forced to divide by the proliferative molecule Shh, secreted by Purkinje neurons (Dahmane and Ruiz i Altaba, 1999; Wallace, 1999; Wechsler-Reya and Scott, 1999). It is believed that the prolonged mitotic activity of the GCPs, consequent to hyperactivation of the Shh pathway, makes them potential targets of transforming insults (Wang and Zoghbi, 2001).

**Figure 1:**
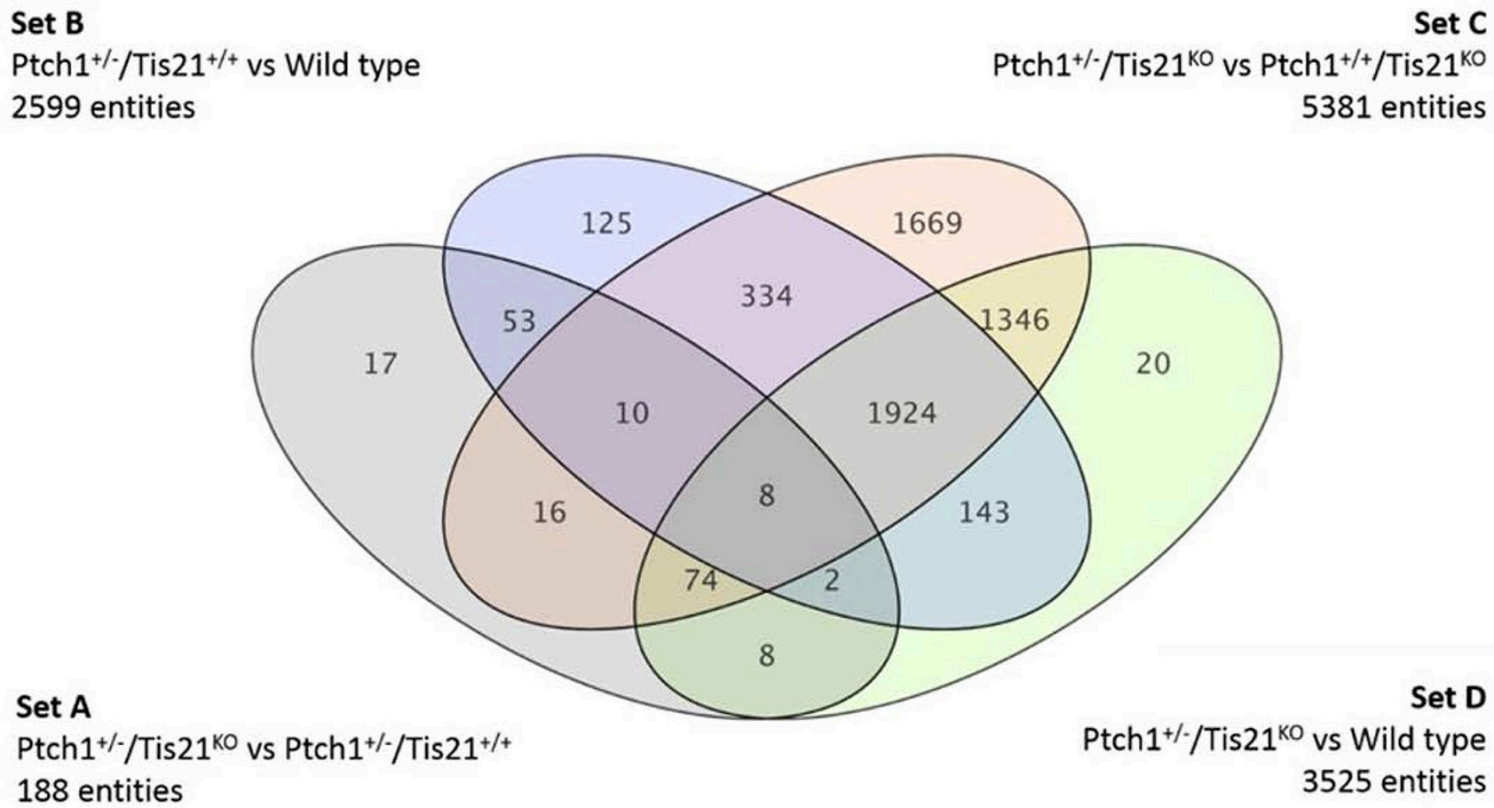
**A Venn diagram showing four genotype pairwise comparisons and the intersection of their differentially expressed gene/sequences set A–D**. Set A corresponds to the pairwise comparison *Ptch1*^+/−^*/Tis21*^*KO*^ vs. *Ptch1*^+/−^*/Tis21*^+/+^; Set B refers to *Ptch1*^+/−^*/Tis21*^+/+^ vs. wild type; Set C concerns *Ptch1*^+/−^*/Tis21*^*KO*^ vs. *Ptch1*^+/+^*/Tis21*^*KO*^; Set D represents the double-knockout contribution in background wild type.

We have previously shown that mice lacking one allele of *Ptch1*, which develop MB with low frequency as result of the activation of the Shh pathway (Hahn et al., 1998), when crossed with mice knockout for the MB suppressor *Tis21* develop MB with very high frequency (Farioli-Vecchioli et al., 2012a,b). We identified as responsible for this effect a defect of migration of the GCPs that, remaining for a longer period in the EGL under the proliferative influence of Shh, developed tumor more frequently. Whole-genome analyses of expression and function indicated that the key molecule responsible for the lack of migration of GCPs is the chemokine Cxcl3 (Farioli-Vecchioli et al., 2012a). Together with *Cxcl3*, we identified other 187 gene sequences, 163 of which have a functional product, whose expression in double mutant *Ptch1* heterozygous/*Tis21* knockout mice was modified, relative to *Ptch1* heterozygous mice in *Tis21* wild-type background (single mutants; Farioli-Vecchioli et al., 2012a). The set of genes whose expression significantly differs in the comparison *Ptch1*^+/−^*/Tis21* wild-type vs. *Ptch1*^+/−^*/Tis21*^*KO*^ will be hereafter defined as Set A (Figure 1).

Here, we aimed to expand the functional investigation of the previous whole-genome analysis of gene expression alterations occurring at the onset of tumorigenesis in the GCPs, in order to further examine the set of genes whose expression is modified in *Ptch1* heterozygous/*Tis21* knockout double mutant mice relative to *Ptch1* heterozygous/*Tis21* wild-type mice (Set A).

Given that *Tis21* mutation has a strong tumorigenic effect in *Ptch1* heterozygous background, with a high increase of MB frequency, we assumed that the transcriptional changes occurring in the Set A of 163 genes after *Tis21* ablation in *Ptch1* background were at the origin of the increased tumorigenicity observed. These genes, referred to as *Tis21*-dependent—given that their expression is by definition modified by the ablation of *Tis21* in *Ptch1* heterozygous background—will be divided in up-regulated and down-regulated, relative to *Ptch1* heterozygous/*Tis21* wild-type mice. It is worth noting that among the genes in Set A whose expression is down-regulated abound those with tumor-inhibitory activity (e.g., *Pag1, PadI4, Lats2*, and *Cxcl3*), while among the up-regulated genes are present tumor facilitators (e.g., *Rab18, Dek*).

Phenotypically, our genomic data refer to GCPs at a very early pre-neoplastic stage, having been isolated from 7 day-old mice, i.e., when the neoplastic lesions have not yet emerged.

The data analyzed interestingly lead to: (i) a link between the Shh signaling and the impairment of the GCPs migration, through a Shh-dependent deregulation of the receptor-mediated endocytosis pathway; (ii) a possible lineage shift of Shh-type GCPs toward retinal precursor phenotype/toward the neural cell type involved in group 3 MB; (iii) the identification of a subset of putative drug targets for MB involved in the regulation of cell cycle, Pdgf, Rapamycin target protein 1 and Hippo signaling pathways. Finally, our findings indicate a role of *Tis21* in the regulation of gene expression, through epigenetic and RNA processing mechanisms.

## Materials and methods

### Gene expression array

Genome-wide expression study design and experimental procedures, of GCPs isolated from the EGL of P7, mice were previously performed with Whole Mouse Genome Microarrays (Agilent Technologies), as described in Farioli-Vecchioli et al. (2012a). GCPs were isolated from Ptch1 heterozygous/Tis21 knockout double mutant and Ptch1 heterozygous/Tis21 wild-type mice of either sex (Farioli-Vecchioli et al., 2012a). In order to extract the mRNA from GCPs for microarray analysis, for each of the four genotypes were used 4 replicates of GCPs isolated from 3-4 mice each, for a total of about 64 mice (Farioli-Vecchioli et al., 2012a). The experiments and all animal procedures were completed in accordance with the current European (directive 2010/63/EU) Ethical Committee guidelines and approved by the Ethical Committee of the Italian Ministry of Health (authorized protocol number 14/2009 dated 14/12/2009, expiry date 14/12/2012, according to Law Decree 116/92). Experiments performed after 14/12/2012 are authorized by the Ethical Committee of the Italian Ministry of Health by protocols 307/2013-B and 193/2015-PR expiring 30/03/2020.

### Functional data analysis

Raw data from microarrays experiments were processed and analyzed using GeneSpringGX 12.5 (Agilent Technologies), as already described (Farioli-Vecchioli et al., 2012a). Pathway enrichment analysis of Set B and Set D genes was performed with MetaCore™ by Thomson Reuters (Ekins et al., 2007). Pathways with corrected enrichment *p*-value *p* < 0.05 were considered significant. MetaCore™ integrated software for functional analysis and its manually curated database have also been used for functional annotation of Tis21-dependent Set A genes, together with DAVID Bioinformatics Resources version 6.7 public database by the National Center for Biotechnology Information (NIH) (Huang da et al., 2009a,b), Mouse Genome Database (MGD) by The Jackson Laboratory, Bar Harbor, Maine (Blake et al., 2014), Cerebellar Development Transcriptome Data Base (CDT-DB) by NIJC, RIKEN-BSI, Japan (Sato et al., 2008) and Universal Protein Resource (UniProt) by the UniProt Consortium (Consortium, 2014).

### Drug target analysis

To identify potential drug targets among the Set A differentially expressed genes, we have used the drug target selection tool via gene list analysis by MetaCore™ (Thomson Reuters) (Ekins et al., 2007) and Thomson Reuters Cortellis Drug Viewer tool (also available on MetaCore™ platform) via pathway analysis. The search has been performed among human primary/direct (Table 3) and secondary/indirect (Table 4) drug targets (see OrthoDB Kriventseva et al., 2015, for the comparison between human and mouse orthologs). The drugs have been taken into account for their targets and not for their use, so not only anti-neoplastic agents are listed in Tables 3–6. Cortellis™ drugs results were compared with records contained in public databases such as DrugBank version 4.2 (Knox et al., 2011; Law et al., 2014), PubChem Compound by NIH (Bolton et al., 2010) and Naturally Occurring Plant based Anticancerous Compound-Activity-Target (Mangal et al., 2013). Finally, to further annotate Set A list genes with respect to known drug-gene interactions and potential druggability, in both mouse and human, we have used the search tools on The Drug Gene Interaction Database (DGIdb) (Griffith et al., 2013) via gene list (Figure 3, Tables 4, 5).

## Results and discussion

### Whole-genome expression changes underlying *Tis21*-dependent activity in GCPs during cerebellum development

By using oligonucleotide microarrays, we monitored the transcriptomic profiles belonging to GCPs isolated at postnatal day 7 (P7), i.e., cells under the proliferative and tumorigenic influence of Shh deregulated signaling in EGL. When expression profiles of genes from either Ptch1 heterozygous GCPs in Tis21 wild-type background (*Ptch1*^+/−^*/Tis21*^+/+^) or double mutant (*Ptch1*^+/−^*/Tis21*^*KO*^) GCPs were compared with the control wild-type (*Ptch1*^+/+^*/Tis21*^+/+^), a consistent subset of genes showed a significant change in expression level, i.e., 2599 in *Ptch1*^+/−^*/Tis21*^+/+^ (Figure 1 Set B) and 3525 in *Ptch1*^+/−^*/Tis21*^*KO*^ (Figure 1 Set D). Instead, the contribution of *Ptch1*^+/−^ in *Tis21* Knockout background was exemplified by 5381 differentially expressed genes (Figure 1 Set C; *Ptch1*^+/−^*/Tis21*^*KO*^ vs. *Ptch1*^+/+^*/Tis21*^*KO*^).

Here we analyze and discuss mainly those genes that were differentially expressed in the pairwise comparison *Ptch1*^+/−^*/Tis21*^*KO*^ vs. *Ptch1*^+/−^*/Tis21*^+/+^ (Set A; Figure 1; Supplementary Table 1), to identify the contribution by *Tis21* in *Ptch1* heterozygous background. These genes are critical as they underlie the great increase of MB frequency observed in *Ptch1* heterozygous mice ablated of *Tis21* (*Ptch1*^+/−^*/Tis21*^*KO*^), relative to *Ptch1* heterozygous mice in a wild-type background (*Ptch1*^+/−^*/Tis21*^+/+^). *Tis21*-dependent mechanisms underlying the onset of Shh-type MB in GCPs during pre-neoplastic development involve a set of 188 sequences (Figure 1 Set A). Among them, about 170 encode for proteins with a known function. In particular, 13 genes belonging to a subset of set A (Figure 1) showed a change of expression that was influenced exclusively by the ablation of *Tis21*: *Tigar, Dsc2, Padi4, Serbp1, Lnx1, Pag1, Olfr670, Mcemp1, Cldn22, Slc25a15, Pth, Pdgfd* and *Cxcl3*.

The validation of some of these genes has already been performed by quantitative real-time PCR (Farioli-Vecchioli et al., 2012a).

### Functional analysis of Ptch1 heterozygous/Tis21-null mouse model deregulated genes

**Figure 2:**
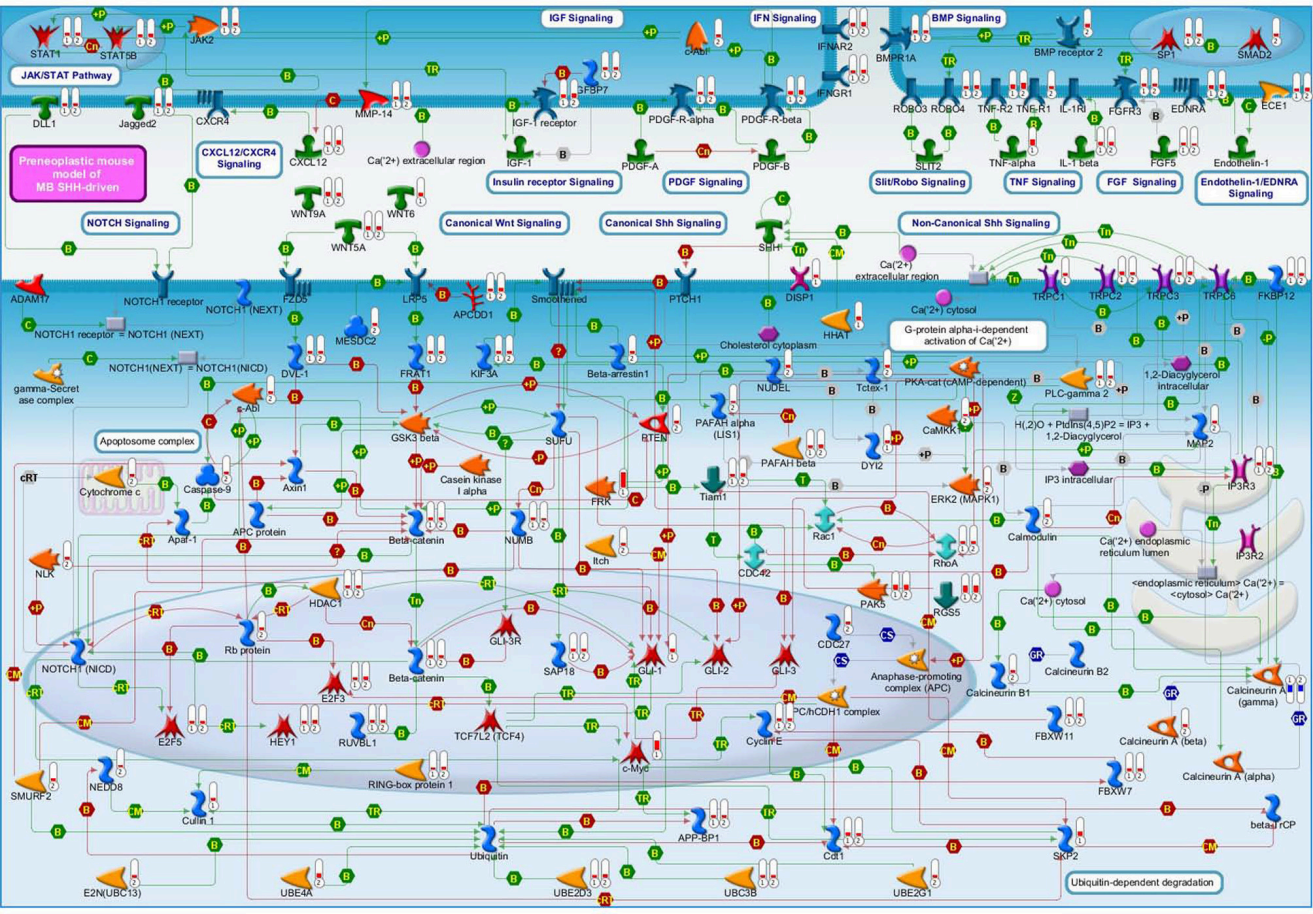
**A molecular overview of our Shh-deregulated mouse model, characterized by the genetic disruption of hedgehog signaling resulting from a heterozygous mutation in the negative regulators ***Ptch1***, whose effects are enhanced by the homozygous deletion of the ***Tis21*** tumor suppressor gene**. The figure shows the proteins mainly involved in neural developmental pathways, encoded by the differentially expressed genes of set B and set D and shown in their subcellular compartments. Each differentially expressed pathway object is labeled with a thermometer that indicates the gene expression changes: downward thermometers have a blue color and indicate down-regulated expression, whereas upward thermometers have a red color and indicate up-regulated expression. The thermometer number 1 is related to the pairwise comparison *Ptch1*^+/−^*/Tis21*^+/+^ vs. wild type Set B, while the thermometer number 2 is related to the pairwise comparison *Ptch1*^+/−^*/Tis21*^−/−^ vs. wild type Set D. Signaling cross-talks between developmental pathways involved in pre-neoplastic GCPs development are initiated by different growth factors and cytokines, the most part of which interacts with G-protein coupled receptors. This is supported by a strong up-regulation of many members of heterotrimeric G-proteins, their target molecules and regulators (data not shown in figure). Among those reported in literature as involved in MB (Roussel and Hatten, 2011), developmental signaling pathways appear to be up-reregulated in our experimental data, according to the far fewer percentages of negative signature genes (Chen et al., 2013) and the over-representation of WNT and axonal guidance genes present in human MB Shh-type (Northcott et al., 2011). Interestingly, a β-Catenin-Gli1 balanced interaction has been recently reported to regulate Shh-driven MB tumor growth in *Ptch1* heterozygous mice *in vitro* (Zinke et al., 2015), while Sox7, a transcription factor that is known to reduce Wnt/β-Catenin stimulated transcription in a dose-dependent manner (Takash et al., 2001), is up-regulated in Set B and *Tis21* ablation enhances its up-regulation in Set D (data not shown in figure). Furthermore, Smo-dependent non-canonical Shh pathways (Jenkins, 2009), which have been reported to modulate cytoskeleton-dependent processes and fluctuation of Ca^2+^ through the plasma membrane in mammalian neurons (Brennan et al., 2012) and suggested in possible association with MB (Briscoe and Therond, 2013), are put in light here for the first time as related to the MB Shh-type mouse model. Evidences of a deregulated Slit-Robo pathway, which is implicated in neuronal migration (Wong et al., 2001; Marillat et al., 2004), are present in our data with the up-regulation of the axon guidance receptor *Robo4*. The ligand of Robo4, Slit2, has been linked to the inhibition of MB cell invasion (Werbowetski-Ogilvie et al., 2006). Proteins belonging to the ubiquitin-dependent degradation of GCPs cell cycle regulators [24] have their genes up-regulated in our model, in particular a number of ubiquitin-conjugating enzymes and some constituents of the SCF (Skip1, Cullin1, F-box)-E3 ubiquitin ligase complex. Among them, a substrate recognition component of the SCF-type E3 ubiquitin ligase, the F-box protein Fbw7, which has been linked to a premature migration of GCPs in conditional *Fbw7*-knockout mice [30]. An up-regulation genes coding for proteins involved in palmitoylation (i.e., HHAT) and transport of Shh (i.e., DISP1) is noticed in in Set D, where Ptch1 sterol-sensing domain seems to control Smoothened activity through Ptch1 vesicular trafficking [34]. Retinoblastoma-associated protein (Rb), as well as its downstream effectors E2F3 and E2F5, has its correspondent gene up-regulated in set D, where the deregulation of the Rb/E2F tumor suppressor complex in MB Shh-driven has been already associated to the E2F1-dependent regulation of lipogenic enzymes in primary cerebellar granule neuron precursors (Bhatia et al., 2011). Figure 4 below shows the set of symbols whereby network objects and interactions between objects are indicated in this figure.

Deregulated genes in our pre-neoplastic model mainly belong to developmental pathways that affect different cellular processes such as cell cycle regulation, proliferation, cell adhesion, cytoskeleton remodeling, apoptosis, survival and differentiation (Table 1). In particular, genes belonging to developmental signaling cascades, differentially expressed in our Shh-deregulated model, depend on the Ptch1 mutation contribution as inferred by set B vs. set D data analysis (Figure 2). As well known in the literature, in fact, developmental cascades, when deregulated, acquire oncogenic effect. Neuronal development and tumorigenesis rely on cell communication via identical signaling pathways, resulting in a complex signaling network that creates a breeding ground for tumor-initiating events (Peifer and Polakis, 2000; Schwartz and Ginsberg, 2002; Clark et al., 2007; Katoh, 2007; Neth et al., 2007; Guo and Wang, 2009; Rodini et al., 2010; Mimeault and Batra, 2011; Roussel and Hatten, 2011; Akhurst and Hata, 2012; Manoranjan et al., 2012).

**Table 1:** **The most informative deregulated genes belonging to the Set A and associated with the influence of Tis21 gene in background ***Ptch1*** heterozygous (GCPs at P7)**.

**Process**	**Text type**	**Down-regulated in Set A**	**Up-regulated in Set A**	**Enrichment probability**
Cell Cycle	NS	Wtap, Sik2, Rab11fip4, Lats2, Zc3h12d, Sema4b, Tigar	Pa2g4, Srpk2, Eif3a, Eif3c, Eif2c1, Taok2, Mphosph10, Rrp1, Ipo7, Taf7, Cdc27, Ckap5	0.000004
Cytoskeleton	NS	Cdc42bpb, Sik2	Ehbp1, Akap2, Rab18, Ckap5, Emd	1
Protein Ubiquitination	NS	Lnx1, Nfx1	Ube2o, Cdc27, Smurf2, Usp36	0.03787
Cell Proliferation	NS	Pag1, Gcnt3, Sema4b	Agtr2, Eif2c1, Gtpbp4, Rps12, Slc6a6	0.69694
Apoptotic Process	NS	Tigar, Ppp1r13l, Serpina3g	Vdac1, Ripk3, Rbm5, Isoc2b, Sltm, Cxcl12	0.32304
Cell Adhesion	NS	Col4a6, Col23a1, Dsc2, Cldn22, Egflam	–	0.39940
Cell differentiation	NS	Zfhx2os, Dazl	Deptor, Foxf2, Lhx5	0.45675
Primary Cilium	MT	Ccdc157, Ccdc171, Rab11fip2, Rab11fip4, Cxcl3	Syne2, Rgs5	0.00001
Vescicle-mediated transport	MT	Rab11fip4, Rab11fip2, Cxcl3	Ehbp1, Zfyve20, Cxcl12, Sgsm2, Ckap5, Vps35, Rab18, Smurf2	0.00574
Retinal Development	MT	H19, Col4a6, Rab11fip4, Bsn, Efna4, Egflam	Vdac1, Taf7, Emd, 9130401M01Rik, Taok2, Hist3h2ba, Tomm22, Vps35, Slc6a6, Pafah1b1, Akap2, Raly, Rps12, Nlk, Pa2g4, Srpk2, Dgkq, Cdc27, Syne2, Ripk3	0.000000
Developmental Process	SD	Gpr82, Smg1, H19, Mettl14, Fat4, Sema4b, Lats2	Rgs5, Sgsm2, Emd, Rab18, Vps35, Nlk, Gigyf2, Kctd5, Ankrd11, Cxcl12, Pdgfd	0.00007
Cell Migration	MT	Cxcl3, Jmy, Efna4, Timp1, Frk, Prrx1, Rab11Fip2	Cxcl12, Pdgfd, Pafah1b1	0.00008
Epigenetic Modulation	SD	Hist2h2bb, Cbx3, Padi4	Hist3h2ba, Ankrd11, Ankrd24, Ankrd26, Brwd1, Dek, Anp32a, Taf7, Pa2g4, Emd, Ipo7	0.000000
RNA Processing	SD	Wtap, Mettl14	Rbm5, Raly, Srpk2, Ddx23, Dek, Htatsf1	0.00312
Nonsense-mediated mRNA decay	SD	Smg1, Upf3b	–	0.00755
Ribonucleoprotein complex biogenesis and assembly	SD	–	Eif3a, Eif3c, Eif2c1, Rmnd1, Rrp1, Gtpbp4, Rps12, Mphosph10, Ipo7	0.00003
Shh-type MB genes	MT	Gli1^*^	Pdgfd	0.0164
Group 3 MB genes	MT		Nlk, Ppp2r2b^*^, Raf1^*^	0.0161

*The genes are clustered in pathways of involvement, according to the functional analysis methods following described. Many genes overlap different clusters. We divide the genes into two categories: down-regulated and up-regulated, depending on whether the mutation of Tis21 causes in double mutant mice an increase or decrease of expression, respectively, relative to Ptch1 heterozygous/Tis21 wild-type mice*.

* *Genes whose levels are changed in Set A although not significantly accordingly to the statistical analysis performed. The functional classes involved in our study have been discussed in the main text (MT), in the Supplementary Data (SD) or are not shown (NS). The enrichment probability of the deregulated genes in each functional class has been calculated using the Fisher Exact Text*.

At the same time, *Tis21* ablation is responsible for the delayed migration of pre-neoplastic precursors outside the EGL, which corresponds to a delayed cell differentiation and represents the key step for MB Shh-type formation. In fact, where GCPs proliferate for a prolonged period in EGL, they became the target of neoplastic transforming insults (Farioli-Vecchioli et al., 2012a,b).

**Figure 3:**
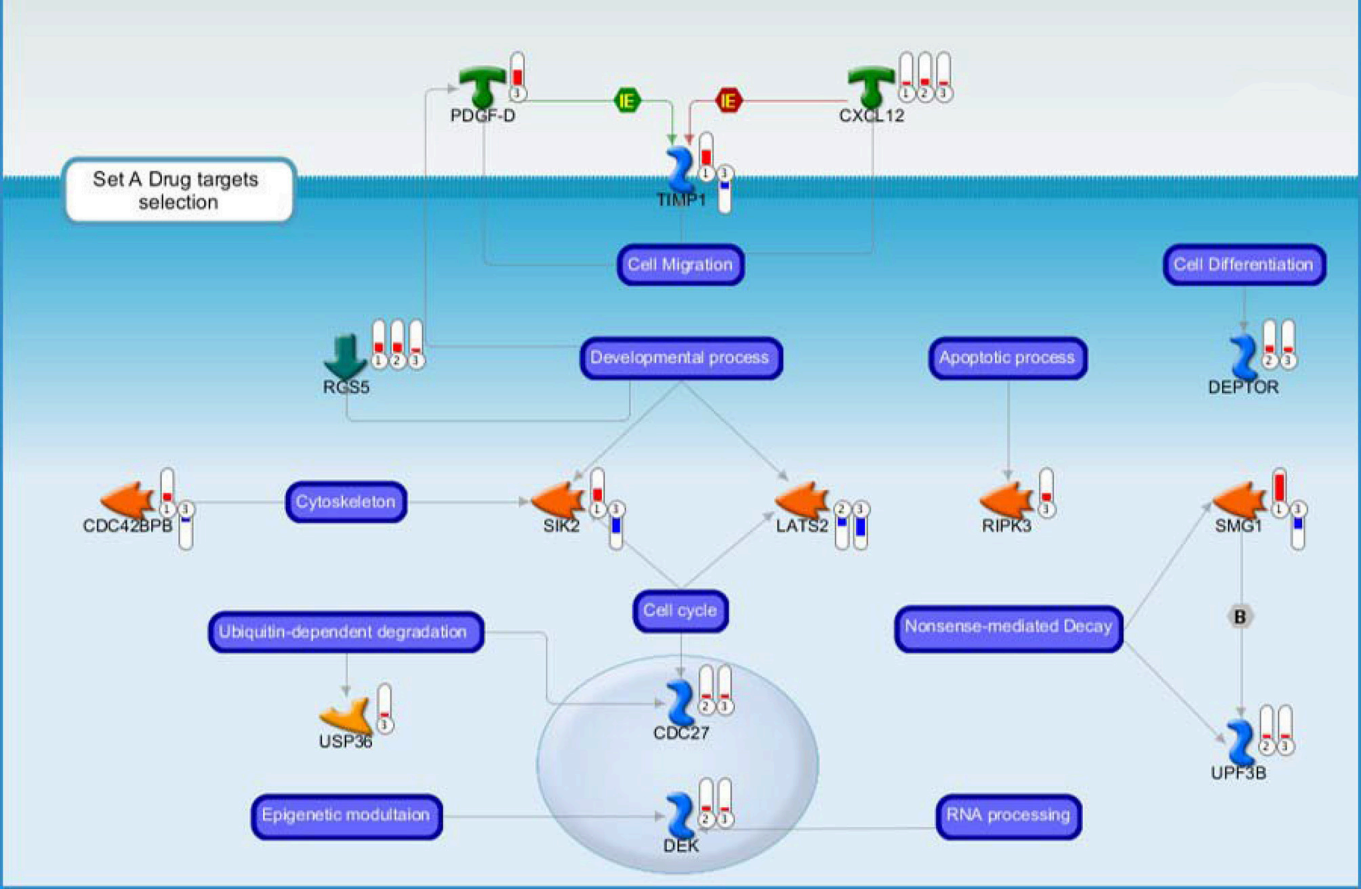
**Drug targets belonging to the Set A discussed in the main text**. Each gene product is labeled with a thermometer indicating the gene expression changes: downward thermometers have a blue color showing down-regulated expression, whereas upward thermometers have a red color showing up-regulated expression. The most part of the figure objects are deregulated also in other two pair comparisons. For this reason, the thermometer number 1 is related to the pairwise comparison *Ptch1*^+/−^*/Tis21*^+/+^ vs. wild type or Set B, the thermometer number 2 is related to the pairwise comparison *Ptch1*^+/−^*/Tis21*^−/−^ vs. wild type or Set D, while the thermometer number 3 is related to the pairwise comparison *Ptch1*^+/−^*/Tis21*^*KO*^ vs. *Ptch1*^+/−^*/Tis21*^+/+^ or Set A. See Figure 4 for the set of symbols, objects and interactions between objects indicated in this figure.

Moreover, we have noticed evidence for the involvement of the primary cilium in our GCPs pre-neoplastic model, mainly in Set B but also in Set A data (Figure 3), and also evidence of Smo-dependent non-canonical Shh pathways. A link between Shh signaling at primary cilium and clathrin-mediated endocytotic trafficking/cytoskeletal remodeling will also be discussed.

**Figure 4:**
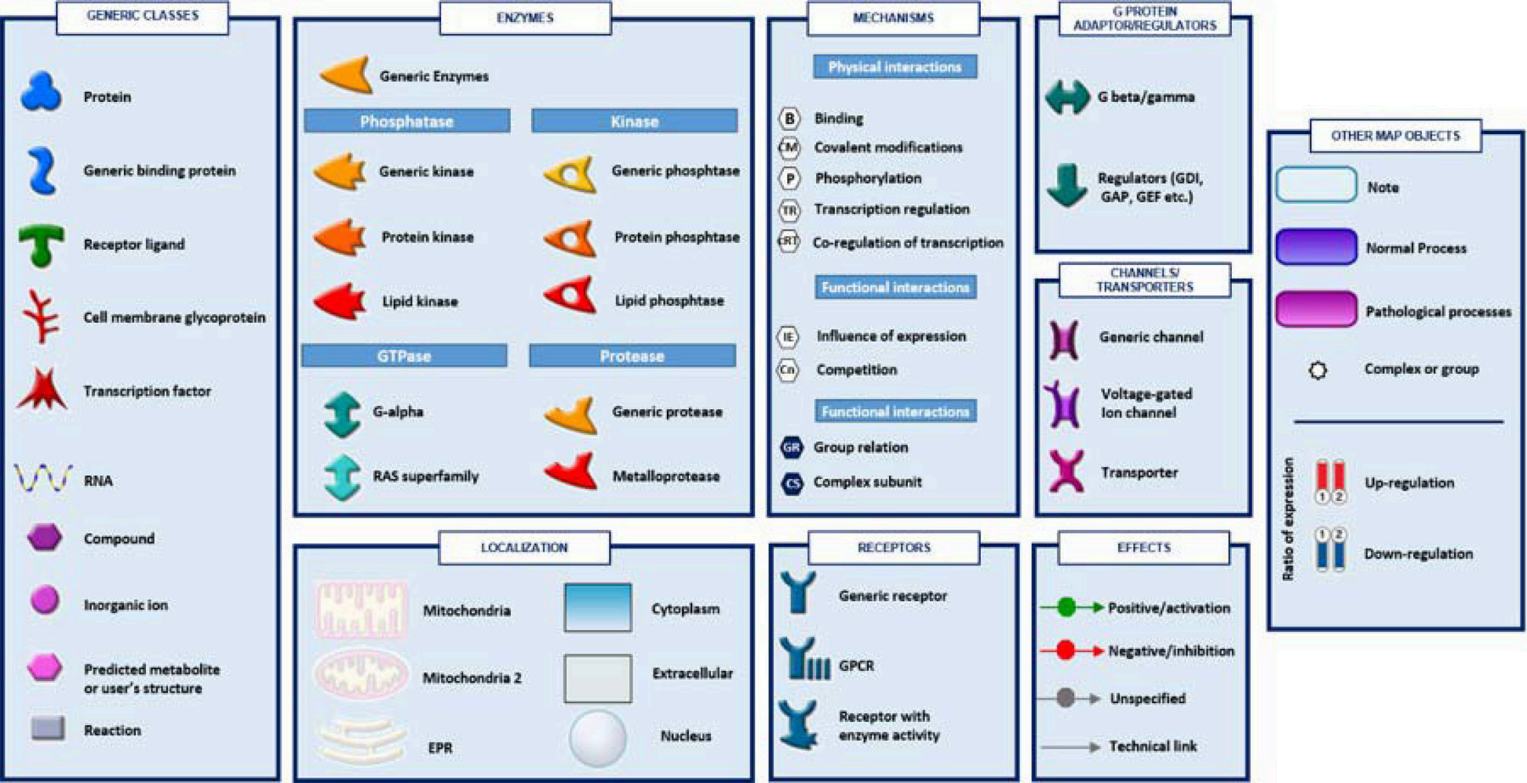
**The figure shows symbols, objects and interactions between objects indicated in Figures 2, 3**.

Another observation is related to the mitogen role of Shh signaling, not only in the developing cerebellum but also in the neuronal tube and overall in the retinal cell specification. In fact, a large number of deregulated genes in our Set A are also involved in the delayed differentiation of retinal cell types. Notably, it has been previously shown a parallelism between MB and retinal development; in fact, the analysis of cell populations in MB-derived from GCPs (particularly the group 3) suggests the occurrence of a potential aberrant trans-lineage differentiation into retinal neuronal precursors (Kool et al., 2008; Hooper et al., 2014). Here, given the large number of genes comprised in set A that is deregulated during the retinal cell development, we want to focus our attention on the timing of exit from the cell cycle, a crucial step in retinal cell development and differentiation, which is under the influence of Shh signaling, as it occurs in the development of MB Shh-type (Dyer, 2004). These considerations will be taken into account for a parallel comparison with our model data.

Finally, a fine regulation at RNA processing, ribosomes and vesicle trafficking level but also an epigenetic modulation were noticed in set A deregulated genes (Table 1). In the following paragraphs, we will discuss the most informative deregulated coding genes belonging to the set A according to their functional clusters (Figure 3 and Table 1), the most of which overlap different clusters. Among them, there is a large number of already known genes as oncogenes and tumor suppressors. In doing this, we have also taken into account the genes which are deregulated in consequence of the *Ptch1* heterozygous.

#### Primary cilium roles in GPCs proliferation and differentiation

Primary cilia are sensory non-motile microtubule-based organelles (Lee and Gleeson, 2010) protruding from the surface of GCPs in the EGL at early post-natal stages (Del Cerro and Snider, 1972), whose requirement for Shh-induced expansion and cerebellar development has been proved using mutants of genes involved in the ciliary formation and maintenance (Chizhikov et al., 2007; Spassky et al., 2008). Among them, the genetic ablation of primary cilia by removing *Kif3a* (which encodes the microtubule plus end-directed kinesin motor 3A protein), blocked MB formation driven by a constitutively active Smoothened protein (Han et al., 2009). Therefore, *Kif3a* down-regulation blocks MB Shh-type formation in a primary cilia-dependent manner; moreover, its activity is not required for GCPs differentiation (Chizhikov et al., 2007). In our model, we observe that *Kif3a* is up-regulated in *Ptch1* heterozygous mice, irrespective of the presence or absence of *Tis21*, which is therefore not involved in the Kif3a-dependent phenotype (Figure 2). This is consistent with the finding that Kif3a is required for the proliferation of the GCPs (Chizhikov et al., 2007) and with our observation that *Tis21* in cerebellum regulates the migration of the GCPs but not their proliferation, while the opposite occurs for *Ptch1*.

Nevertheless, in our model, several genes encoding for the coiled-coil domain containing proteins are deregulated in Set A, and thus are dependent on *Tis21*, i.e., *Ccdc157* and *Ccdc171* (Table 1). One-fourth of the deregulated genes in Set A corresponds to coiled-coils proteins (data not shown), whose highly versatile protein folding motif is related to different biological processes, from subcellular infrastructure maintenance to trafficking control (Burkhard et al., 2001; Rose et al., 2005) and cilia-related (McClintock et al., 2008; Munro, 2011). A coiled-coil containing protein is also Rab11 family-interacting protein 4 encoded by *Rab11fip4* (Muto et al., 2006, 2007), whose role in our model will be discussed more in detail in other paragraphs together with the functional product of *Rab11fip2*, and their wide implication in Shh signaling at primary cilium as a protein involved in microtubule-based vesicle trafficking. Another protein, Nesprin-2 encoded by *Syne2*, is known to mediate centrosome migration and is essential for early ciliogenesis and formation of the primary cilia by the interaction with the coiled-coil domain of Meckelin protein (Dawe et al., 2009). Notably, *Ccdc157, Ccdc171, Rab11fip2*, and *Rab11fip4* are significantly down-regulated in Set A, while *Syne2* is up-regulated. Also a novel repressor of hedgehog signaling, whose gene *Rgs5* is up-regulated in set A, has been proven to be present with Smo in primary cilia (Mahoney et al., 2013). This would suggest that *Tis21*-dependent tumorigenesis in a (proliferation-independent) way involves ciliogenesis. This latter may be also enhanced by *Syne2* after *Tis21* ablation.

Evidences of direct involvement of Shh signaling on the increase of Ca^2+^ levels (Ca^2+^ spikes) have been shown at the primary cilium of chicken embryonic spinal neurons. In this system has been observed that Shh (a recombinant N-terminal molecule) may recruit second messengers (i.e., calcium—Ca^2+^—and inositol triphosphate) by a non-canonical pathway, through the activation of the Smoothened protein, which translocates to the cilium and becomes activated by phosphorylation at its C-terminal from a G-protein-coupled receptor kinase 2 (Riobo et al., 2006; Belgacem and Borodinsky, 2011; Brennan et al., 2012). Belgacem and Borodinsky (2011) proposed a model in which the primary cilium acts as a subcellular compartment for Shh signaling allowing the spatiotemporal integration of the second messengers through a Smoothened-dependent recruitment of G_i_ proteins and Phospholipase C that in turn increases inositol triphosphate levels. The opening of Inositol triphosphate receptors-operated stores and the following activation of Transient receptor potential cation channel 1 (Trpc1) results in an increased Ca^2+^ spike activity. This model could fit with our data (Figure 3) in which *Plc-gamma2, Ip3r3, Trpc1, Trpc2*, and *Trpc3* are up-regulated in *Ptch1* heterozygous mice (but independently from *Tis21*). Notably, this is the first time that such regulation is observed directly in a mouse model of Shh-type MB, as it had been previously only suggested (Briscoe and Therond, 2013). Furthermore, the authors suggested that the Smoothened-dependent Ca^2+^ spike activity is necessary for Shh-induced differentiation of spinal postmitotic neuron. Moreover, the role of second messenger signaling in the regulation of cerebellar granule cell migration has been studied in different mouse models (Komuro et al., 2015), which highlighted the direct evidence of the role of Ca^2+^ signaling in granule cell turning and modulation of their migration rate. The revision of these studies, performed by Komuro et al. (2015), suggested the role of Ca^2+^ as potential therapeutic target for some deficits in granule cell migration since its downstream effectors control the assembly and disassembly of cytoskeletal elements.

In the last years, the discovery of the role of the primary cilium in Shh signaling captured the attention of the scientific community, leading to test a large number of molecules that modulate SMO cilial translocation acting on different therapeutic potential targets in different types of cancer among which MB (Amakye et al., 2013). Loss of cilia in cancer has been suggested to be responsible for an insensitivity of cancer cells to environmental repressive signals, based in part on derangement of cell cycle checkpoints governed by cilia and centrosomes (Plotnikova et al., 2008). The importance of the role of cilia in Shh-driven medulloblastoma allografts derived from *Ptch1*^+/−^*P53*^−/−^ mice has been shown using a Shh antagonist, i.e., arsenic trioxide (a therapeutic agent for acute promyelocytic leukemia), which inhibits the growth of tumor through the prevention of Shh ciliary accumulation and the reduction of the stability of the Gli2 transcriptional effector (Kim et al., 2010).

#### Receptor-mediated endocytosis mechanisms, microtubule-based vesicle recycling and intracellular membrane trafficking

**Figure 5:**
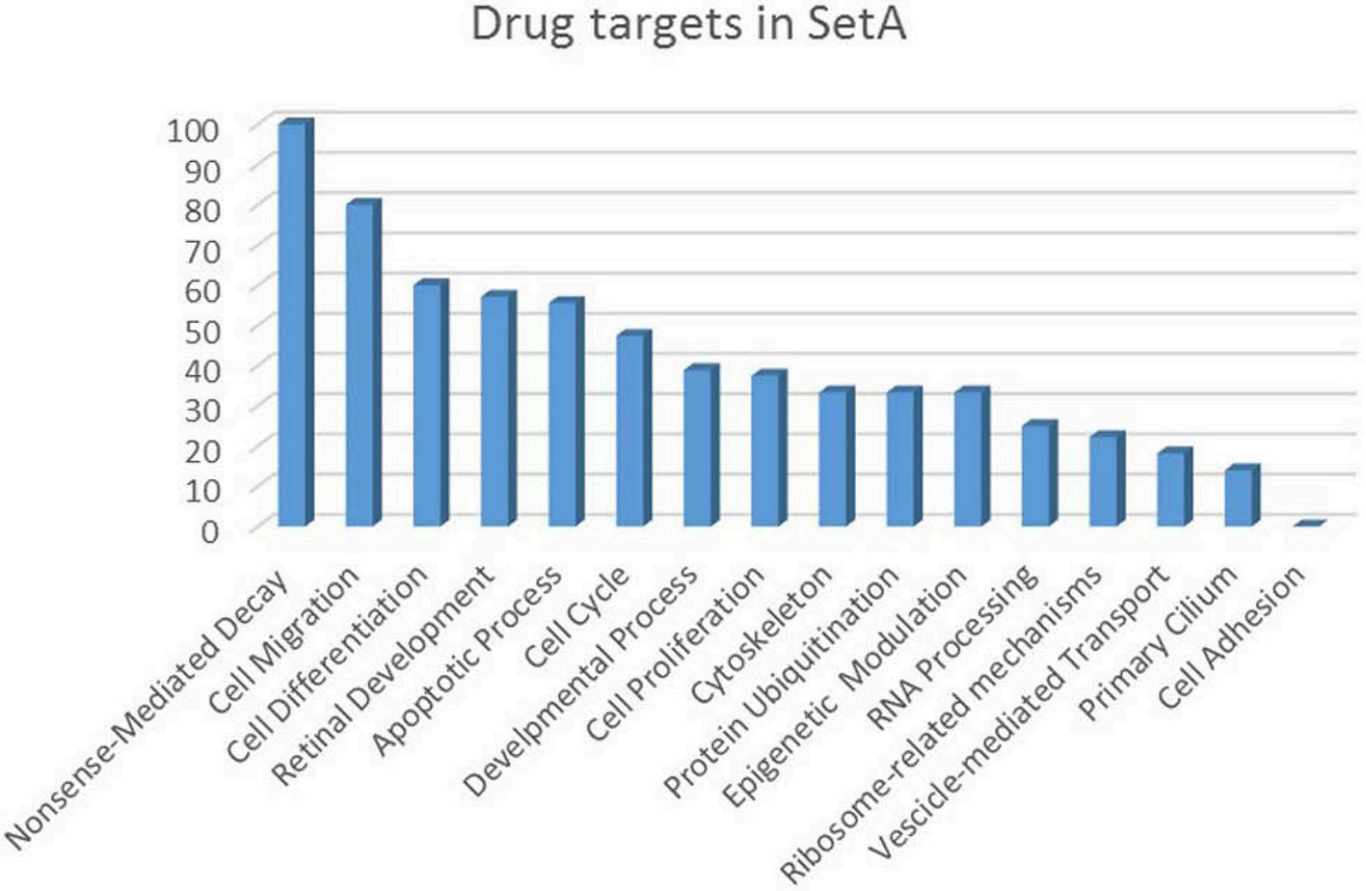
**Drug targets percentage per functional class of the Set A deregulated genes**. The drug targets identified in Set A are showed as percentage with respect to the functional classes to which they belong.

Other genes deregulated in Set A are involved in endocytic trafficking clathrin-dependent (Figure 5), and a certain number is related to cytoskeletal remodeling and primary cilium that could be very interesting for their implications for target therapy.

The clathrin-dependent endocytic mechanism is a receptor-mediated endocytosis type, which involves clathrin-coated vesicles, early endosomes, microtubule-based vesicle trafficking, lysosomes and recycling transport vesicles in its pathway (Le Roy and Wrana, 2005). Evidences of a deregulated clathrin-mediated endocytosis pattern have been reported for MB group 4 during early human neurogenesis (Hooper et al., 2014), and detected in our Set B (data not shown) as well as in Set A, where a large number of deregulated genes belongs to this signaling: *Rab11fip4* (Horgan and McCaffrey, 2009), *Ehbp1* (Guilherme et al., 2004), *Rab11fip2* (Fan et al., 2004), *Zfyve20* (de Renzis et al., 2002; Naslavsky et al., 2004), *Cxcl12* (Fan et al., 2004; Teicher and Fricker, 2010; Zhu et al., 2012), *Sgsm2* (Yang et al., 2007), *Ckap5* (Foraker et al., 2012), *Vps35* (Foraker et al., 2012), and *Rab18* (Foraker et al., 2012).

*Rab11fip4* is known to interact with Rab11 (a Rab GTPase involved in the regulation of intracellular membrane trafficking), which in turn interacts with Rab-coupling protein, a protein involved in the formation of lamellipodia, which facilitates invasive cancer cell migration (Kelly et al., 2012). *Ehbp1* gene product is involved in actin reorganization coupled to endocytosis clathrin-mediated (Guilherme et al., 2004). *Zfyve20* is known to regulate structure, sorting, endocytic/recycling pathway of the early endosomal compartment by the interaction with Rab4 and Rab5 (de Renzis et al., 2002; Naslavsky et al., 2004). *Vps35*, among other genes involved in intracellular membrane trafficking, is deregulated in Set A. It encodes for the vacuolar protein sorting-associated protein 35, a component of the trimeric cargo-selective retromer complex, required for the efficient endosome-to-Golgi recycling of many membrane proteins among which Wntless. It is also known to regulate endosomal tubule dynamics (Harbour et al., 2010; Berwick and Harvey, 2014). The oncogene *Rab18* is required for normal ER structure and the regulation of endocytic traffic (Lütcke et al., 1994; Gerondopoulos et al., 2014). This Rab GTPasi is also known as a MB antigen (Behrends et al., 2003). Interestingly, Rab18 has been localized at lipid droplets (LDs) level (Martin et al., 2005), and its overexpression causes close apposition of LDs to membrane cisternae connected to the rough ER (LD-associated membrane or LAM) (Ozeki et al., 2005). Indeed, *de novo* lipid synthesis has been found in certain highly proliferative and aggressive tumors such as MB Shh-type, suggesting that the Shh-E2F1-FASN axis regulating *de novo* lipid synthesis in cancers could be used as therapeutic target in hedgehog-dependent tumors (Bhatia et al., 2011). Remarkably, *Rab11fip2* encodes for a Rab11 family-interacting protein 2, which directly interacts with the actin-based myosin Vb motor protein regulating Cxcr2 recycling and receptor-mediated chemotaxis; hence, Rab11fip2 links Rab11 to molecular motor proteins and cell migration (Jones et al., 2006; Horgan and McCaffrey, 2009). Moreover, the passage of the internalized Cxcr2 through the Rab11-recycling system appears to have a key role in the physiological response to a chemokine that follows to the formation of clathrin-coated vesicles; in fact, Rab11 family-interacting protein 2 can form a complex with AP-2 that is a major clathrin adaptor complex in cells (Cullis et al., 2002; Fan et al., 2004; Le Roy and Wrana, 2005). Furthermore, the Rab11 involvement into the regulation of vesicular trafficking during primary ciliogenesis has been already put in light (Knödler et al., 2010; Hsiao et al., 2012). A consequence of the above findings, indicating an interaction between Cxcr2 and the clathrin pathway, is that, since Cxcl3 binds the Cxcr2 receptor (Zlotnik et al., 2006), we can infer that this chemokine receptor-mediated chemotaxis mechanism is clathrin-dependent and linked through Rab11fip2 to the primary cilium, in which the Shh signaling takes part. Other evidence of an involvement of Rab11fips and Shh signaling derives from Rab11fip4 (see the retina development section). This protein seems to be involved in the regulation of the membrane trafficking system through interaction with other small GTPases, among which could be Ras-related protein Rab-23, and in the negative regulation of Shh signaling at the primary cilium (Muto et al., 2007; Hsiao et al., 2012).

All this points to an important link between Shh signaling, operating through the primary cilium, and GCPs impaired cell migration, through a clathrin-Cxcl3-Cxcr2-mediated chemotaxis and microtubule-based endocytic vesicle recycling trafficking. Furthermore, the findings of a study about the role of endosomes around mother centriole appendages, and their Rab11-dependent recycling activity that requires centrosome-associated endosome proteins (Hehnly et al., 2012), seem to be in line with our data (see drug target section). Interestingly, they showed that (i) the appendages of the mother centriole and recycling endosomes are in intimate contact, as first evidence for a novel centrosome-anchored molecular pathway and regulation of endosome recycling; (ii) there is a structural association between the endosome and the centrosome with new and unexpected implications for recycling endosome functions, such us that one related to cilia formation; (iii) it is also possible that Rab11, and other endosome-associated molecules bound to the centrosome, may play dual roles in endosome and centrosome function (Hehnly et al., 2012).

#### Retinal development

In mice, retinal development occurs between E11.5 and P8, as uncommitted neuroblasts leave the cell cycle and commit to retinal cell fates (Mu et al., 2001). Thanks to mice models, it is known that aberrant proliferation during the development of the neural tube, of cerebellum and retina, leads to embryonal and early postnatal tumors (Dyer, 2004). The potent mitogen Shh positively controls the proliferation of their neuronal precursor cells (Martí and Bovolenta, 2002). In particular, Shh signaling plays a pivotal role in regulating the proliferation of retinal progenitor cells (RPCs) and the differentiation of retinal ganglion cells (RGCs) during vertebrate retinal development, acting in a cell-specific manner; namely, in mouse Shh is required as positive regulator of RPCs proliferation and as negative regulator of RGCs production, by inhibiting cell-cycle exit (Wang et al., 2005; Wallace, 2008).

A further molecular target which may be responsible for the regulation of retinal cell proliferation and hence for cancer cell proliferation was suggested to be Rb; in fact, the levels of Rb protein appear critical for the development of retinal tumors (Sicinski et al., 1995). The rationale for this is that Rb, when active, inhibits the cycle at the G1 checkpoint, prior to cell differentiation, whereas its inactivation, exerted by phosphorylation from cyclinD1/CDK4, is known to start the cell cycle progression. Thus, high levels of Rb may be more difficult to inactivate and vice-versa, thus critically linking the Rb-dependent developmental regulation of proliferation during neurogenesis to cancer cell proliferation for certain types of tissues (Sicinski et al., 1995). Indeed, in our model we observe that in GCPs heterozygous for *Ptch1* (set B: *Ptch1*^+/−^*/Tis21*^+/+^ vs. *Ptch1*^+/+^*/Tis21*^+/+^) cyclin D1 expression does not change while Rb does increase. This suggests that in Shh-driven neoplastic GCPs the increase of Rb protein is compensatory.

In the case of MB, a parallel between developmental neurobiology and oncology was suggested for the proliferating progenitor cells of the retina and cerebellar granule neurons, where the failure to exit the cell cycle leads to aberrant cell proliferation during development in mice (Romer and Curran, 2004). Notably, MB-types Cluster D and E (also referred to as groups 4 and 3, respectively) have been found to be marked by a deregulated expression of retinal photoreceptor genes suggesting a distinct origin (i.e., non-cerebellar) from stem cells during the embryonic development, with respect to MB Shh-type in human (Kool et al., 2008).

In this context, concerning the contribution of Tis21 deletion to the MB development, in Set A we noticed a great number of deregulated genes that have been previously described as involved in retinal development. This comparison could be useful to suggest some common mechanisms related to the progenitor cells cell-cycle exit failure. A consistent subset of Set A genes has been previously described as being involved in cellular expression patterns of mouse early retinal development; these gene were previously recognized by analyzing the outer retinal neuroblastic layer, which in early developmental stages consists almost entirely of mitotic progenitor cells: *Vdac1, Taf7, Emd, 9130401M01Rik, Taok2, Hist3h2ba, Tomm22, Vps35, H19, Slc6a6, Pafah1b1, Akap2, Raly, Rps12, Nlk, Pa2g4* and *Srpk2* (Blackshaw et al., 2004). These genes are all up-regulated in Set A except for *H19*. Furthermore, a number of other genes that were identified in other studies as being involved in retinal development, are down-regulated in set A: *Col4a6* (Bai et al., 2009), *Rab11fip4* (Muto et al., 2007), *Bsn* (Dick et al., 2003), *Efna4* (Marcus et al., 1996; Poliakov et al., 2004; Triplett and Feldheim, 2012), *Egflam* (whose product is also known as Pikachurin) (Omori et al., 2012); conversely, other retinal genes up-regulated in set A are: *Dgkq* (Pilz et al., 1995), *Cdc27* (Leung et al., 2008), *Syne2* (Yu et al., 2011), *Slc6a6* (Vinnakota et al., 1997; Warskulat et al., 2007), *Ripk3* (Trichonas et al., 2010).

The genes listed above belong to different functional clusters, and some of them will be discussed more in detail in their paragraph of pertinence. Interestingly, the mouse *Rab11fip4*, whose product regulates the Rab GTPases and is predominantly expressed in the developing neural tissues, among which retina, acts as regulator of RPCs cell-cycle exit and their subsequent differentiation (Muto et al., 2007). Rab11fip4 seems to be involved in the regulation of membrane trafficking system through interaction with other small GTPases and in the negative regulation of Shh signaling (Muto et al., 2007). Moreover, the *Syne2* gene product is known to mediate nuclear migration during mammalian retinal development connecting the nucleus with dynein/dynactin and kinesin proteins (Yu et al., 2011).

This comparison is in line with the evidence that progenitors from the developing cerebral cortex, cerebellum and retina share a common expression program, suggesting a common evolutionary origin of the different progenitors cells (Livesey et al., 2004), and implying a possible common differentiation program. Our data suggest that this developmental process, possibly at the steps of migration and differentiation, is regulated by *Tis21*.

#### Migration

The migration of the GCPs is known to be induced by responses to local environmental cues. However, the alterations of migratory behavior may also depend on intrinsic programs (Komuro et al., 2013). In our study, we observed in Set A the differential expression of a great number of genes belonging to migration, cell adhesion and differentiation mechanisms. Among the genes implicated in migration seven are down-regulated after ablation of *Tis21* in Shh-activated background, namely, *Cxcl3, Jmy, Efna4, Timp1, Frk, Prrx1*, and *Rab11fip2*, and three are up-regulated: *Cxcl12, Pdgfd*, and *Pafah1b*.

*Cxcl3* is a chemokine known to have chemotactic activity for neutrophils (Wuyts et al., 1999). Chemotaxis but also angiogenesis seems to be activated by the interaction with its receptor (Addison et al., 2000; Zlotnik et al., 2006). In our previously published data, *Cxcl3* has been identified as a gene whose transcription is regulated positively by Tis21 (which directly associates to the *Cxcl3* promoter), suggesting that the evident role played *in vivo* by *Tis21* as inducer of the migration of the GCPs out of the EGL might occur through its functional product; this implies Cxcl3 as a new pharmacological target for medulloblastoma therapy, also considering that MB lesions were reduced using this chemokine as treatment (Farioli-Vecchioli et al., 2012a,b). Recently, the anti-cancer role of this chemokine has also been recognized for non-small lung cancer, mediated by the activity of interleukin-27 (Airoldi et al., 2015). Jmy, a junction mediating and regulatory protein, is known to induce cell motility by promoting actin assembly and regulating cadherins in the cytoplasm; in DNA damage conditions it undergoes nuclear accumulation, where acts as p53-cofactor promoting apoptosis (Shikama et al., 1999; Coutts et al., 2007; Zuchero et al., 2009). Together, these findings suggest that the ability of Jmy to regulate actin and cadherin, by coordinating cell motility with the p53 response, could underlie a common pathway (Coutts et al., 2009). Furthermore, actin assembly has been found to regulate the nuclear import of Jmy in response to DNA damage (Zuchero et al., 2012). Thus, an abnormal activity and/or localization of Jmy could contribute to tumor invasion, thus making this gene as a potential tumor therapeutic target (Wang, 2010). In theory, the down-regulation of *Jmy* may contribute to enhance the rate of MB in our mouse model, possibly by controlling the migration of the GCPs as shown for Cxcl3. Regarding *Efna4*, this gene encodes for the EphrinA4 protein that has been linked with the migration of neuronal cells during development (Poliakov et al., 2004). A recent study showed that EphrinA4 and the Ephrin type-B receptor 4 are almost exclusively expressed in Shh MBs, while other Ephrins are expressed in non–Shh MBs; furthermore, a strategy of overexpression and silencing, applied specifically to EphrinB1, showed its ability to control the migration of MB cell lines (McKinney et al., 2015). Further evidence of the migration-promoting activity of EphrinA4 has been observed in human glioma cells, where it acts by accelerating a canonical FGFR signaling pathway (Fukai et al., 2008). *Timp1* protein product is a metalloproteinase inhibitor known to be involved in cell adhesion and migration of human neural stem cells (hNSCs). In fact, through its cell surface receptor CD63, TIMP-1 activates β1 integrin, FAK (focal adhesion kinase) and the PI3K (phosphoinositide 3-kinase) signal transduction pathway, resulting in the migration of hNSCs (Lee et al., 2014). Due to its chemoattractant properties, TIMP-1 has been identified in the same study as a therapeutic target for human glioma. The *Frk* gene product is a Src kinase known as Tyrosine-protein kinase FRK, which controls the migration and invasion of human glioma cells by regulating JNK/c-Jun signaling (Zhou et al., 2012). Moreover, the Tyrosine-protein kinase FRK acts as a tumor suppressor in breast cancer by regulating the stability of PTEN, as the loss of *Rak* (i.e., *Frk*) induced tumorigenicity in immortalized normal mammary epithelial cells (Yim et al., 2009). In mouse brain, *Pten* is known to be expressed starting at approximately postnatal day 0 (Lachyankar et al., 2000) and has also been correlated with the regulation of neuronal precursor cell migration (Li et al., 2002). In Set B Pten is up-regulated. The paired-related homeobox transcription factor 1, which is encoded by *Prrx1*, is an epithelial-mesenchymal transition (EMT) inducer in embryos, where this process is required for the formation of tissues for which cells originate far from their final destination (Ocaña et al., 2012). EMT is modified and exploited by cancer cells for metastatic dissemination and also in cancer cells. In particular, the loss of *Prrx1* has been related to the ability of cancer cells to acquire tumor-initiating abilities concomitantly with stem cells properties (Ocaña et al., 2012). Furthermore, paired-related homeobox transcription factor 1 has been found to promote tenascin-C–dependent fibroblast migration when its expression was induced by Focal adhesion kinase 1 (McKean et al., 2003). *Fak* is up-regulated in both Set B and Set D. Very interesting is the down-regulation of *Rab11fip2*, whose role in the endocytic recycling pathway has been linked to cell migration (Jones et al., 2006) as previously discussed (Section Receptor-Mediated Endocytosis Mechanisms, Microtubule-Based Vesicle Recycling and Intracellular Membrane Trafficking).

Among the genes up-regulated in Set A related to migration there is *Cxcl12*, which encodes for a deeply studied chemokine involved in different mechanisms in cancer development and metastatic invasion (Duda et al., 2011; Hattermann and Mentlein, 2013), but also described as involved in the migration of neuronal cells through both its receptor, C-X-C chemokine receptor type 4 and Atypical chemokine receptor 3 (Tiveron and Cremer, 2008; Memi et al., 2013; Yang et al., 2013). Cxcl12 appears to exert an action opposite to Cxcl3, as it promotes the localization of the GCPs to the EGL by chemoattraction, being released from meninges (Klein et al., 2001; Zhu et al., 2002). Thus, the upregulation of *Cxcl12*, consequent to the ablation of *Tis21*, synergizes with the downregulation of *Cxcl3* in preventing the migration of the GCPs from the EGL. Notably, the chemoattraction of cerebellar granule cells by Cxcl12, whose receptor C-X-C chemokine receptor type 4 is coupled to a G protein, is selectively inhibited by the soluble EphB receptor; this inhibition is blocked by a truncated PDZ-RGS3 lacking the RGS domain, which activates the G-proteins. Therefore, this points to the existence of a pathway connecting B ephrins and Cxcl12 to the regulation of G protein–coupled chemoattraction, and leads to a model for regulation of migration in cerebellar development (Lu et al., 2001). In this regard, in our model (Set A) we have detected not only a down-regulation of *Efna4*, which is a cell surface GPI-bound ligand for Eph receptors, but also the up-regulation of a regulator of heterotrimeric G protein signaling, i.e., *Rgs5*. Therefore, in our model the decrease of *Cxcl3* and *Efna4* and the increase of *Cxcl12* after ablation of *Tis21* in *Ptch1* heterozygous mice would synergize in impairing the migration of GCPs from the EGL. As mentioned above, the increase of *Cxcl12* in the *Tis21*-null EGL GCPs would prevent their migration by chemoattraction (Zhu et al., 2002). Moreover, the PDGFR pathway members have been recently studied in correlations with MB Shh-driven and MB cell migration, and an upregulation of PDGFRA (receptor for PDGF-A), PDGF-D (ligand for PDGFRB) and Cxcl12 has been observed in Shh-type MB (vs. non Shh MBs). In particular, the activation of the PDGFR pathway has been shown to activate the Cxcl12 receptor (i.e., CXCR4, via inhibition of GRK6) and thus cell migration (Yuan et al., 2013). A relevant caveat of that study is that the direction of cell migration, relative to the EGL, was not assessed, having the analysis been conducted only *in vitro* (Yuan et al., 2013). In our model, we noticed an up-regulation of *Pdgfd* and *Cxcl12* genes in Set A, while both *Pdgfra* and *Pdgfrb* and also *Cxcl12* are up-regulated in Set B and D (see Figure 3). This indicates that the heterozygosity of *Ptch1* is in itself a condition inducing the expression of both the Cxcr4 and Pdgfr pathways, and that the additional ablation of *Tis21* further enhances their activation. Finally, *Pafah1b1*, up-regulated in Set A, encodes for Lis1, a microtubule regulator that is required for correct neuronal migration during development (Hippenmeyer et al., 2010; Escamez et al., 2012).

#### Epigenetic modulation

The transcriptional reprogramming of the epigenetic patterns is among the causes of tumorigenesis. Nowadays, the epigenetic regulation of transcription and genome organization in MB Shh-type pathogenesis is extensively studied (Batora et al., 2014; Hovestadt et al., 2014; Shi et al., 2014). We have previously provided a first functional genomic analysis of epigenetically regulated genes or interacting proteins for our mouse model *Tis21* KO, identified in background either *Ptch1* heterozygous or wild-type (Farioli-Vecchioli et al., 2012b). We also have recently shown that the Tis21 protein binds to histone deacetylases (HDAC1/4/9) in GCPs, where they are required for the Tis21-dependent inhibition of cyclin D1 expression (Micheli et al., 2016). In the present analysis, a great number of genes encoding proteins involved in epigenetic regulation appear to be deregulated in Set A (three *down-regulated* and 12 *up-regulated)*. They mostly belong to the category of the Histone modification rather than to the Chromatin remodeling one (Table 2), which categories are described in (Arrowsmith et al., 2012; Plass et al., 2013). Our data are in line with those previously published highlighting a great number of deregulated or mutated histone modifiers involved in medulloblastoma (Northcott et al., 2012) and their importance as oncogenes or as tumor suppressors (Cohen et al., 2011). Furthermore, we highlight also the deregulation of five Histone modifier Regulators, up-regulated in Set A, namely: *Emd* (Berk et al., 2013), *Anp32a* (Seo et al., 2001; Fan et al., 2006; Kular et al., 2009), *Taf7* (Gegonne et al., 2001, 2013; Kloet et al., 2012), *Pag2g4* (Zhang et al., 2003), *Ipo7* (Jäkel et al., 1999; Mühlhäusser et al., 2001). A detailed description of Set A genes involved in epigenetic modulation is presented in Supplementary Data, at section “Epigenetic modulation.”

**Table 2:** **Regulators of the epigenome identified in Set A**.

**Functional Groups**	**Subgroups**	**Down-regulated in Set A**	**Up-regulated in Set A**
Histone modification	Histones	Hist2h2bb (Marzluff et al., 2002; González-Romero et al., 2010)	Hist3h2ba (Marzluff et al., 2002; González-Romero et al., 2010)
	Writers	Cbx3 (Takanashi et al., 2009)	-
	Editors	Padi4 (Wang et al., 2004, 2012; Chang et al., 2009; Tanikawa et al., 2012; Christophorou et al., 2014; Deplus et al., 2014)	Ankrd11 (Behrends et al., 2003; Zhang et al., 2004; Plass et al., 2013; Gallagher et al., 2015), Ankrd24 (Plass et al., 2013), Ankrd26 (Plass et al., 2013)
	Readers	Cbx3 (Arney and Fisher, 2004)	Brwd1 (Huang et al., 2003; Bai et al., 2011; Arrowsmith et al., 2012; Filippakopoulos and Knapp, 2012; Plass et al., 2013)
Chromatin remodeling	Nucleosome remodeling factor	Padi4 (Christophorou et al., 2014)	-
	Chromatin remodeling factor	-	Dek (Cavellán et al., 2006; Hu et al., 2007; Privette Vinnedge et al., 2013; Saha et al., 2013; Hooper et al., 2014)

*Histones and enzymes involved in histone modification and chromatin remodeling are showed in the table. They have been classified in subgroups according to their function and divided in enzymes that transfer the modification or writers, enzymes that modify or revert a modification also called editors or erasers and enzymes that mediate the interactions of proteins or protein complexes with the modification known as readers (Arrowsmith et al., 2012; Plass et al., 2013)*.

#### Drug targets

The increasing recognition of the heterogeneity of molecular basis underlying cancer allows the identification and development of molecularly targeted agents and their transfer to the patients (Rask-Andersen et al., 2011; Saletta et al., 2014). Here we provide a drug target identification through the genomic analysis of deregulated MB Shh-type *Tis21* knockout-dependent genes in Set A and, where possible, the identification of potentially druggable targets (Figure 3, Tables 3–5), performed using the methods described in the appropriate section. This analysis has given as a result 20 primary, 31 secondary, 53 druggable targets and 18 gene targets among Set A elements (Tables 3–6). Their distribution within the functional classes is showed in Figure 5. and the putative drug targets identified in SetA, here discussed, are shown in Figure 3.

**Table 3:** **A list containing the direct targets and their drugs obtained using MetaCore™ (i.e., ATP1A1) and Cortellis™ db**.

**MetaCore™/Cortellis™ Therapeutic Drug-target direct Interactions**				
**Gene Symbol**	**Target**	**Drug**	**Effect**	**Pubmed**
ATP1A1	ATP1A1	Trichlormethiazide intracellular	Inhibition	
		Istaroxime	Inhibition	12904068, 12388640, 17239702
HSD11B2	11 beta-Hydroxysteroid dehydrogenase	Enoxolone	Inhibition	8504732, 16012754, 16309197, 16391818, 17566745, 18721818, 18600055, 19949310, 24613819
H19	H19	BC-822	Inhibition	
PDGF	PDGF	CR002	Inhibition	17308324, 16510766, 12937299
AGTR2	AGTR2	XR-510	Inhibition	
		PD-123319	Inhibition	
		MP-157	Inhibition	
		EMA401	Inhibition	23489258
ACACA	Acetyl-CoA carboxylase 1 isoform 1	CP-640186	Inhibition	12842871, 15341732, 23358327, 15934915
		CP-610431	Inhibition	12842871
SRPK2	SRPK2	SRPIN340	Inhibition	16840555, PMC2916360
GBF1	GBF1	Brefeldin A	Inhibition	3192548, PMC160661, 2004424

*The Cortellis™ drugs list has been compared and expanded using others public databases as described in Materials and Methods section*.

**Table 4:** **A list containing the indirect drug-target interactions obtained using MetaCore™**.

**MetaCore**™**Secondary Drug-target Interactions**				
**Drug**	**Gene Symbol**	**Target**	**Effect**	**Pubmed**
Lestaurtinib extracellular region	CDC42BPB	CDC42BPB	Inhibition	
	NLK	NLK	Inhibition	
	LATS2	LATS2	Inhibition	
	FRK	FRK	Inhibition	
	TAOK2	TAO2	Inhibition	
	SRPK2	SRPK2	Inhibition	
	SIK2	QIK	Inhibition	
Midostaurin intracellular	LATS2	LATS2	Unspecified	19654408
	FRK	FRK	Unspecified	19654408
	SRPK2	SRPK2	Unspecified	19654408
	SIK2	QIK	Unspecified	19654408
Ca('2+) cytosol	DGKB	Diacylglycerol kinase, beta	Activation	9003401, 16288460, 10206945, 12832407
	PADI4	PAD4	Activation	11798170, 15247907
	DGKA	DGK-alpha	Activation	9003401, 16288460, 10206945, 12832407
	DGKG	Diacylglycerol kinase, gamma	Activation	9003401, 16288460, 10206945, 12832407
	VDAC1	VDAC 1	Activation	12022949, 16597621
Estradiol extracellular region	BTG2	BTG2	Inhibition	12959972
	RGS5	RGS5	Inhibition	17374850
	CXCL12	SDF-1	Activation	17362937, 17555868
	HSD11B2	HSD11B2	Inhibition	9623596, 10503719
Dabrafenib intracellular	FRK	FRK	Inhibition	
	TAOK2	TAO2	Inhibition	
	SIK2	QIK	Inhibition	
Vandetanib intracellular	CDC42BPB	CDC42BPB	Unspecified	22037378
	FRK	FRK	Unspecified	22037378
	SIK2	QIK	Inhibition	22037377, 22037378
Tozasertib intracellular	TAOK2	TAO2	Unspecified	22037378
	SRPK2	SRPK2	Unspecified	18183025,22037378
	SIK2	QIK	Unspecified	18183025,22037378
Retinoic acid extracellular region	PADI4	PAD4	Activation	10488123
	ADAMTS5	Aggrecanase-2	Activation	17255106
Uprosertib intracellular	NLK	NLK	Inhibition	
	FRK	FRK	Inhibition	
Dexamethasone extracellular region	SLC25A15	SLC25A15 (ORC1)	Activation	12055339, 11997094
	HSD11B2	HSD11B2	Activation	16872738
Zn('2+) nucleus	ZNF286A	ZNF286A	Unspecified	11347906
	BSN	Bassoon	Unspecified	9806829, 9679147, 9679147
Pazopanib extracellular region	FRK	FRK	Unspecified	22037378
	TAOK2	TAO2	Unspecified	22037378
Masitinib intracellular	FRK	FRK	Inhibition	22037378
Pyridoxal Phosphate intracellular	SLC25A15	SLC25A15 (ORC1)	Inhibition	12807890
	ACACA	ACACA	Inhibition	16249179
Fedratinib intracellular	TAOK2	TAO2	Inhibition	
	SIK2	QIK	Inhibition	
Nilotinib intracellular	CDC42BPB	CDC42BPB	Unspecified	22037378
	FRK	FRK	Inhibition	22037378
Epigallocatechin-3-gallate extracellular region	CXCL1	GRO-1	Inhibition	
TAK-901 intracellular	FRK	FRK	Inhibition	
	SIK2	QIK	Inhibition	
INK128 intracellular	FRK	FRK	Inhibition	
	SIK2	QIK	Inhibition	
Ibrutinib intracellular	FRK	FRK	Inhibition	
Zn('2+) extracellular region	ADAMTS10	ADAM-TS10	Activation	11867212
	ADAMTS5	Aggrecanase-2	Activation	11867212
TG100115 intracellular	LATS2	LATS2	Unspecified	
Perphenazine extracellular region	ATP1A1	ATP1A1	Unspecified	6167716
Pamapimod intracellular	NLK	NLK	Unspecified	
Eprosartan extracellular region	AGTR2	AGTR2	Inhibition	2016730, 8515425
Aclarubicin intracellular	TIMP1	TIMP1	Activation	12118337
Promazine extracellular region	ATP1A1	ATP1A1	Unspecified	6167716
Phenoxybenzamine extracellular region	ATP1A1	ATP1A1	Inhibition	2576298
Carbenoxolone intracellular	HSD11B2	HSD11B2	Inhibition	10566667, 10664531, 12190302
Anisindione intracellular	ACACA	ACACA	Unspecified	4067989
Maprotiline extracellular region	ATP1A1	ATP1A1	Inhibition	2576298
Irbesartan extracellular region	AGTR2	AGTR2	Inhibition	2329553
Prazepine extracellular region	ATP1A1	ATP1A1	Inhibition	2576298
lorcainide extracellular region	ATP1A1	ATP1A1	Inhibition	10593659
Pirfenidone intracellular	TIMP1	TIMP1	Inhibition	17086734
NO extracellular region	SLC6A6	SLC6A6	Activation	11698241, 14992266, 15166008
Taurine extracellular region	SLC6A6	SLC6A6	Inhibition	9130593, 10936171, 12437590
Fenoprofen intracellular	ACACA	ACACA	Inhibition	1347398
Meloxicam extracellular region	TIMP1	TIMP1	Inhibition	16536903
Promethazine extracellular region	ATP1A1	ATP1A1	Unspecified	6167716
Biotin intracellular	ACACA	ACACA	Activation	12421859, 14749229, 15539280
Furosemide intracellular	HSD11B2	HSD11B2	Inhibition	9724039
Dimethyl sulfoxide intracellular	PADI4	PAD4	Activation	10488123
Thiram intracellular	HSD11B2	HSD11B2	Inhibition	12901862
Disopyramide extracellular region	ATP1A1	ATP1A1	Inhibition	10593659
Fluoxymesterone intracellular	HSD11B2	HSD11B2	Inhibition	
Digitoxin extracellular region	ATP1A1	ATP1A1	Inhibition	11525233
Methylene blue intracellular	GUCY1A3	GUCY1A3	Inhibition	12031394, 12729841
Heparin extracellular region	CXCL12	SDF-1	Inhibition	10570220, 15741341, 17264079, 17726466
Atenolol extracellular region	ATP1A1	ATP1A1	Inhibition	11201505
Hg('2+) extracellular region	SLC25A15	SLC25A15 (ORC1)	Inhibition	12807890
Procainamide extracellular region	ATP1A1	ATP1A1	Inhibition	10593659
Trazodone extracellular region	ATP1A1	ATP1A1	Inhibition	2576298
Nicotinic acid intracellular	ACACA	ACACA	Inhibition	8857918, 10195935, 11171611, 12873710, 15030302
Imatinib intracellular	FRK	FRK	Unspecified	15711537, 18183025, 22037378
Sotalol extracellular region	ATP1A1	ATP1A1	Unspecified	11201505
Tocainide extracellular region	ATP1A1	ATP1A1	Inhibition	
TRV-120027 extracellular region	AGTR2	AGTR2	Unspecified	
Ruxolitinib intracellular	TAOK2	TAO2	Unspecified	22037378
Ruboxistaurin intracellular	LATS2	LATS2	Unspecified	22037378
Cisplatin extracellular region	ATP1A1	ATP1A1	Inhibition	
Trifluoperazine extracellular region	ATP1A1	ATP1A1	Unspecified	6167716
Doxycycline intracellular	TIMP1	TIMP1	Activation	20874665, 17122880, 17145119, 19442784
Losartan extracellular region	AGTR2	AGTR2	Inhibition	1469703, 1875348, 1920360, 7473594, 7562905, 7606390, 7990105, 8064808, 8246245, 8277505, 8277506, 8340920, 8421274, 11310611, 11749394, 12213451, 16039125, 7990105, 14761190
Silmitasertib intracellular	SRPK2	SRPK2	Inhibition	
Chlorpromazine extracellular region	ATP1A1	ATP1A1	Unspecified	6167716
R547 intracellular	TAOK2	TAO2	Unspecified	22037378
17alpha-Hydroxyprogesterone extracellular region	HSD11B2	HSD11B2	Inhibition	10566667
Alprazolam extracellular region	ATP1A1	ATP1A1	Inhibition	2576298
Digoxin extracellular region	ATP1A1	ATP1A1	Inhibition	11072868
CFI-400945 intracellular	NLK	NLK	Unspecified	
Clotiapine extracellular region	ATP1A1	ATP1A1	Inhibition	2576298
Indenolol extracellular region	ATP1A1	ATP1A1	Inhibition	1685314, 11201505
Testosterone extracellular region	ACACA	ACACA	Activation	16579987
Aldosterone extracellular region	ATP1A1	ATP1A1	Activation	16767692, 10718622
Encainide extracellular region	ATP1A1	ATP1A1	Inhibition	2168576, 10593659
Regorafenib intracellular	FRK	FRK	Inhibition	
Cholic acid intracellular	HSD11B2	HSD11B2	Inhibition	12015312
BMS-387032 intracellular	TAOK2	TAO2	Unspecified	
Angiotensin II extracellular region	AGTR2	AGTR2	Activation	16570916, 2362273
Tivozanib intracellular	FRK	FRK	Inhibition	
Clofilium extracellular region	ATP1A1	ATP1A1	Inhibition	15637453
Amoxapine extracellular region	ATP1A1	ATP1A1	Inhibition	2576298
Tranilast intracellular	TIMP1	TIMP1	Inhibition	16393468
Zn('2+) cytosol	RBM5	RBM5	Unspecified	10352938
Androstanolone extracellular region	RPS12	RPS12	Inhibition	15525599
Ketoconazole intracellular	HSD11B2	HSD11B2	Inhibition	10664531
Thioridazine extracellular region	ATP1A1	ATP1A1	Unspecified	6167716
Retinoic acid intracellular	GCNT3	GCNT3	Activation	15591039
Prochlorperazine extracellular region	ATP1A1	ATP1A1	Unspecified	6167716
Latanoprost extracellular region	TIMP1	TIMP1	Activation	12454040
Piperacillin intracellular	LACTB	LACTB	Inhibition	
Afatinib intracellular	FRK	FRK	Inhibition	
Afuresertib intracellular	NLK	NLK	Inhibition	
Topiramate intracellular	ACACA	ACACA	Activation	16415917
Nebentan extracellular region	AGTR2	AGTR2	Unspecified	
Celiprolol intracellular	ATP1A1	ATP1A1	Inhibition	1685314, 11201505
Disulfiram intracellular	HSD11B2	HSD11B2	Inhibition	12901862
Cimetidine intracellular	PADI4	PAD4	Unspecified	
Candesartan extracellular region	AGTR2	AGTR2	Unspecified	16039125
Progesterone intracellular	HSD11B2	HSD11B2	Inhibition	9623596, 10566667, 10664531, 12915667, 9055382
Loxapine extracellular region	ATP1A1	ATP1A1	Inhibition	2576298
Nadolol extracellular region	ATP1A1	ATP1A1	Inhibition	11201505
Hydrochlorothiazide intracellular	HSD11B2	HSD11B2	Inhibition	10334975
Deoxycholic acid intracellular	HSD11B2	HSD11B2	Inhibition	12015312
Metoprolol extracellular region	ATP1A1	ATP1A1	Inhibition	11201505
D-Glucose extracellular region	SLC6A6	SLC6A6	Inhibition	10516137, 12870156
Paricalcitol	PTH	PTH	Inhibition	9697664, 16797393

*The only exception is the record related to the PTH gene that has been taken from Cortellis Drug Viewer list and compared with others public databases, as described in Materials and Methods section*.

**Table 5:** **A drug-target interactions list and the correspondent source of information obtained using the Search Drug-Target Interactions tool on DGIdb, where a drug-gene interaction is a known interaction between a known drug compound and a target gene**.

**DGIdB Drug-Gene Interactions**					
**Search Term**	**Gene**	**Drug**	**Interaction Type**	**Source**	**Source Version**
**ACACA**	ACACA-acetyl-CoA carboxylase alpha	TOFA	n/a	GuideToPharmacologyInteractions	04-mar-15
		BIOTIN	n/a	DrugBank	3
ADAMTS5	ADAMTS5-ADAM metallopeptidase with thrombospondin type 1 motif, 5		n/a	DrugBank	3
			n/a	DrugBank	3
		BATIMASTAT	n/a	DrugBank	3
AGTR2	AGTR2-angiotensin II receptor, type 2	PD123319	n/a	GuideToPharmacologyInteractions	04-mar-15
		[125I]CGP42112	n/a	GuideToPharmacologyInteractions	04-mar-15
		COMPOUND 21 [PMID: 22802221]	n/a	GuideToPharmacologyInteractions	04-mar-15
		CGP42112	n/a	GuideToPharmacologyInteractions	04-mar-15
		ANGIOTENSIN III	n/a	GuideToPharmacologyInteractions	04-mar-15
		ANGIOTENSIN II	n/a	GuideToPharmacologyInteractions	04-mar-15
		NOVOKININ	n/a	GuideToPharmacologyInteractions	04-mar-15
		ANGIOTENSIN A	n/a	GuideToPharmacologyInteractions	04-mar-15
		CANDESARTAN CILEXETIL	Antagonist	TTD	4.3.02 (2011.08.25)
		TAK-491	Antagonist	TTD	4.3.02 (2011.08.25)
		PS433540	Antagonist	TTD	4.3.02 (2011.08.25)
		AZILSARTAN	Antagonist	TTD	4.3.02 (2011.08.25)
		AZILSARTAN MEDOXOMIL	Antagonist	TTD	4.3.02 (2011.08.25)
		TAK-591	Antagonist	TTD	4.3.02 (2011.08.25)
		TASOSARTAN	Antagonist	DrugBank	3
ATP1A1	ATP1A1-ATPase, Na/K transporting, alpha 1 polypeptide	ALMITRINE	n/a	TEND	01-Aug-2011
		BEPRIDIL	n/a	TEND	01-Aug-2011
		ACETYLDIGITOXIN	n/a	TEND	01-Aug-2011
		OUABAIN	n/a	TEND	01-Aug-2011
		DIGOXIN	n/a	TEND	01-Aug-2011
		DIGITOXIN	n/a	TEND	01-Aug-2011
		DESLANOSIDE	n/a	TEND	01-Aug-2011
		ACETYLDIGITOXIN	n/a	GuideToPharmacologyInteractions	04-mar-15
		DESLANOSIDE	n/a	GuideToPharmacologyInteractions	04-mar-15
		DIGITOXIN	n/a	GuideToPharmacologyInteractions	04-mar-15
		DIGOXIN	n/a	GuideToPharmacologyInteractions	04-mar-15
		OUABAIN	Binder	TTD	4.3.02 (2011.08.25)
		LUMEFANTRINE	Binder	TTD	4.3.02 (2011.08.25)
		CICLOPIROX	Binder	TTD	4.3.02 (2011.08.25)
		ALUMINIUM	Binder	TTD	4.3.02 (2011.08.25)
		CHLOROPROCAINE	Blocker	TTD	4.3.02 (2011.08.25)
		ARTEMETHER	Binder	TTD	4.3.02 (2011.08.25)
		ALMITRINE	Binder	TTD	4.3.02 (2011.08.25)
		DESLANOSIDE	Binder	TTD	4.3.02 (2011.08.25)
		DIGITOXIN	Inhibitor	DrugBank	3
		HYDROFLUMETHIAZIDE	Other/Unknown	DrugBank	3
		BEPRIDIL	Inhibitor	DrugBank	3
		DIGOXIN	n/a	DrugBank	3
		ACETYLDIGITOXIN	Inhibitor	DrugBank	3
		ALUMINIUM	Binder	DrugBank	3
		MAGNESIUM	n/a	DrugBank	3
		ETHACRYNIC ACID	Inhibitor	DrugBank	3
		DIAZOXIDE	Other/Unknown	DrugBank	3
		TRICHLORMETHIAZIDE	Inhibitor	DrugBank	3
		POTASSIUM	n/a	DrugBank	3
		DESLANOSIDE	Inhibitor	DrugBank	3
		ALMITRINE	Binder	DrugBank	3
		OUABAIN	Inhibitor	DrugBank	3
		BRETYLIUM	Inhibitor	DrugBank	3
CEACAM3	CEACAM3-carcinoembryonic antigen-related cell adhesion molecule 3	ARCITUMOMAB	Antibody	TTD	4.3.02 (2011.08.25)
CXCL12	CXCL12-chemokine (C-X-C motif) ligand 12	TINZAPARIN	Binder	DrugBank	3
DEPTOR	DEPTOR - DEP domain containing MTOR-interacting protein	AZD8055	Inhibitor	MyCancerGenome	13-mar-13
		MLN0128	Inhibitor	MyCancerGenome	13-mar-13
		OSI-027	Inhibitor	MyCancerGenome	13-mar-13
		ERLOTINIB	Antagonist	DrugBank	3
FRK	FRK-fyn-related kinase	REGORAFENIB	Inhibitor	MyCancerGenomeClinicalTrial	30-Feburary-2014
HSD11B2	HSD11B2-hydroxysteroid (11-beta) dehydrogenase 2	NADH	n/a	DrugBank	3
LARS	LARS-leucyl-tRNA synthetase	L-LEUCINE	n/a	DrugBank	3
PADI4	PADI4-peptidyl arginine deiminase, type IV	N-[(1S)-1-(AMINOCARBONYL)-4-(ETHANIMIDOYLAMINO)BUTYL]BENZAMIDE	n/a	DrugBank	3
		L-CITRULLINE	n/a	DrugBank	3
PDGFD	PDGFD-platelet derived growth factor D	SUNITINIB	Inhibitor	MyCancerGenomeClinicalTrial	30-Feburary-2014
PPP1R13L	PPP1R13L-protein phosphatase 1, regulatory subunit 13 like	CISPLATIN	n/a	PharmGKB	12-Jul-2012
		CYCLOPHOSPHAMIDE	n/a	PharmGKB	12-Jul-2012
		THALIDOMIDE	n/a	PharmGKB	12-Jul-2012
		PLATINUM COMPOUNDS	n/a	PharmGKB	12-Jul-2012
		PLATINUM	n/a	PharmGKB	12-Jul-2012
PTH	PTH-parathyroid hormone	NORLEUCINE	n/a	DrugBank	3
SLC25A15	SLC25A15-solute carrier family 25 (mitochondrial carrier; ornithine transporter) member 15	L-ORNITHINE	n/a	DrugBank	3
SRPK2	SRPK2-SRSF protein kinase 2	PHOSPHOAMINOPHOSPHONIC ACID-ADENYLATE ESTER	n/a	DrugBank	3
		PURVALANOL	n/a	DrugBank	3
		ADENINE	n/a	DrugBank	3
		ADENOSINE-5′-DIPHOSPHATE	n/a	DrugBank	3
TAOK2	TAOK2-TAO kinase 2	PHOSPHONOSERINE	n/a	DrugBank	3
VDAC1	VDAC1-voltage-dependent anion channel 1	DIHYDROXYALUMINIUM	Inhibitor	DrugBank	3

**Table 6:** **A sub-list of Set A genes with the correspondent selected druggable gene category and source of information obtained using the Search Druggable Gene Categories tool on DGIdb, where a druggable gene category is a group of genes that are thought to be potentially druggable by various methods of prediction**.

**DGIdB Druggable Targets**			
**Search term**	**Gene**	**Druggable Gene Category**	**Sources**
ACACA	ACACA-acetyl-CoA carboxylase alpha	DRUGGABLE GENOME	HopkinsGroom RussLampel
		DRUG RESISTANCE	GO
ADAMTS10	ADAMTS10-ADAM metallopeptidase with thrombospondin type 1 motif, 10	PROTEASE	GO dGene
		NEUTRAL ZINC METALLOPEPTIDASE	GO
		DRUGGABLE GENOME	RussLampel dGene
ADAMTS5	ADAMTS5-ADAM metallopeptidase with thrombospondin type 1 motif, 5	DRUGGABLE GENOME	RussLampel dGene
		PROTEASE	GO dGene
		NEUTRAL ZINC METALLOPEPTIDASE	GO
AGTR2	AGTR2-angiotensin II receptor, type 2	G PROTEIN COUPLED RECEPTOR	dGene GO HopkinsGroom
		DRUGGABLE GENOME	dGene RussLampel HopkinsGroom
AKAP2	AKAP2-A kinase (PRKA) anchor protein 2	KINASE	GO
ATP1A1	ATP1A1-ATPase, Na/K transporting, alpha 1 polypeptide	DRUGGABLE GENOME	RussLampel HopkinsGroom
		TRANSPORTER	HopkinsGroom GO
		ION CHANNEL	HopkinsGroom
		ABC TRANSPORTER	GO
		DRUG RESISTANCE	GO
BTG2	BTG2-BTG family, member 2	DNA REPAIR	GO
CDC27	CDC27-cell division cycle 27 homolog (S. cerevisiae)	TUMOR SUPPRESSOR	GO
CDC42BPB	CDC42BPB-CDC42 binding protein kinase beta (DMPK-like)	DRUGGABLE GENOME	RussLampel dGene HopkinsGroom
		SERINE THREONINE KINASE	dGene GO
		KINASE	GO HopkinsGroom
CXCL1	CXCL1-chemokine (C-X-C motif) ligand 1	GROWTH FACTOR	GO
CXCL12	CXCL12-chemokine (C-X-C motif) ligand 12	CELL SURFACE	GO
		EXTERNAL SIDE OF PLASMA MEMBRANE	GO
		GROWTH FACTOR	GO
DAZL	DAZL-deleted in azoospermia-like	TUMOR SUPPRESSOR	GO
DEK	DEK - DEK oncogene	DNA REPAIR	GO
DGKQ	DGKQ-diacylglycerol kinase, theta 110 kDa	KINASE	GO
		TRANSCRIPTION FACTOR BINDING	GO
DPP10	DPP10-dipeptidyl-peptidase 10 (non-functional)	DRUGGABLE GENOME	RussLampel HopkinsGroom dGene
		PROTEASE	HopkinsGroom GO dGene
EFNA4	EFNA4-ephrin-A4	KINASE	GO
		TYROSINE KINASE	GO
ERG	ERG-v-ets erythroblastosis virus E26 oncogene homolog (avian)	CLINICALLY ACTIONABLE	FoundationOneGenes
FOXF2	FOXF2-forkhead box F2	TRANSCRIPTION FACTOR COMPLEX	GO
		TRANSCRIPTION FACTOR BINDING	GO
FRK	FRK-fyn-related kinase	KINASE	GO HopkinsGroom
		TYROSINE KINASE	GO dGene
		DRUGGABLE GENOME	HopkinsGroom dGene RussLampel
GPR82	GPR82-G protein-coupled receptor 82	G PROTEIN COUPLED RECEPTOR	dGene GO HopkinsGroom
		DRUGGABLE GENOME	dGene RussLampel HopkinsGroom
GTPBP4	GTPBP4-GTP-binding protein 4	TUMOR SUPPRESSOR	GO
GUCY1A3	GUCY1A3-guanylate cyclase 1, soluble, alpha 3	DRUGGABLE GENOME	HopkinsGroom RussLampel
HSD11B2	HSD11B2-hydroxysteroid (11-beta) dehydrogenase 2	DRUGGABLE GENOME	RussLampel HopkinsGroom
		DRUG RESISTANCE	GO
		SHORT CHAIN DEHYDROGENASE REDUCTASE	HopkinsGroom
IPO7	IPO7-importin 7	TRANSPORTER	GO
JMY	JMY-junction mediating and regulatory protein, p53 cofactor	TUMOR SUPPRESSOR	GO
		DNA REPAIR	GO
LARS	LARS-leucyl-tRNA synthetase	DRUGGABLE GENOME	HopkinsGroom RussLampel
LATS2	LATS2-LATS, large tumor suppressor, homolog 2 (Drosophila)	SERINE THREONINE KINASE	GO dGene
		KINASE	GO HopkinsGroom
		TUMOR SUPPRESSOR	GO
		DRUGGABLE GENOME	HopkinsGroom dGene RussLampel
NAPEPLD	NAPEPLD - N-acyl phosphatidylethanolamine phospholipase D	PHOSPHOLIPASE	GO
NLK	NLK-nemo-like kinase	KINASE	HopkinsGroom GO
		DRUGGABLE GENOME	HopkinsGroom dGene RussLampel
		SERINE THREONINE KINASE	dGene GO
		TRANSCRIPTION FACTOR BINDING	GO
PA2G4	PA2G4-proliferation-associated 2G4, 38 kDa	DRUGGABLE GENOME	RussLampel HopkinsGroom
		TUMOR SUPPRESSOR	GO
		PROTEASE	HopkinsGroom
PAFAH1B1	PAFAH1B1-platelet-activating factor acetylhydrolase 1b, regulatory subunit 1 (45 kDa)	TUMOR SUPPRESSOR	GO
PDGFD	PDGFD-platelet-derived growth factor D	GROWTH FACTOR	GO
PPP1R13L	PPP1R13L-protein phosphatase 1, regulatory subunit 13 like	TRANSCRIPTION FACTOR BINDING	GO
PTH	PTH-parathyroid hormone	HORMONE ACTIVITY	GO
RBM5	RBM5-RNA binding motif protein 5	DRUGGABLE GENOME	HopkinsGroom RussLampel
RIPK3	RIPK3-receptor-interacting serine-threonine kinase 3	DRUGGABLE GENOME	RussLampel dGene
		SERINE THREONINE KINASE	GO dGene
		KINASE	GO
SENP7	SENP7-SUMO1/sentrin specific peptidase 7	PROTEASE	dGene GO
		DRUGGABLE GENOME	dGene
SIK2	SIK2-salt-inducible kinase 2	SERINE THREONINE KINASE	GO dGene
		KINASE	GO HopkinsGroom
		DRUGGABLE GENOME	RussLampel HopkinsGroom dGene
SLC25A15	SLC25A15-solute carrier family 25 (mitochondrial carrier; ornithine transporter) member 15	TRANSPORTER	GO
		DRUGGABLE GENOME	RussLampel
SLC6A6	SLC6A6-solute carrier family 6 (neurotransmitter transporter, taurine), member 6	DRUGGABLE GENOME	RussLampel HopkinsGroom
		TRANSPORTER	GO HopkinsGroom
SMG1	SMG1-smg-1 homolog, phosphatidylinositol 3-kinase-related kinase (C. elegans)	PHOSPHATIDYLINOSITOL 3 KINASE	HopkinsGroom
		DRUGGABLE GENOME	HopkinsGroom RussLampel dGene
		SERINE THREONINE KINASE	dGene GO
		KINASE	GO
		DNA REPAIR	GO
SRPK2	SRPK2-SRSF protein kinase 2	SERINE THREONINE KINASE	GO dGene
		KINASE	GO
		TUMOR SUPPRESSOR	GO
		DRUGGABLE GENOME	RussLampel dGene
SYT11	SYT11-synaptotagmin XI	TRANSPORTER	GO
TAF7	TAF7-TAF7 RNA polymerase II, TATA box binding protein (TBP)-associated factor, 55 kDa	TRANSCRIPTION FACTOR COMPLEX	GO
		HISTONE MODIFICATION	GO
		TRANSCRIPTION FACTOR BINDING	GO
TAOK2	TAOK2-TAO kinase 2	KINASE	HopkinsGroom GO
		DRUGGABLE GENOME	HopkinsGroom RussLampel dGene
		SERINE THREONINE KINASE	dGene GO
		TUMOR SUPPRESSOR	GO
TIMP1	TIMP1-TIMP metallopeptidase inhibitor 1	PROTEASE INHIBITOR	GO dGene
		DRUGGABLE GENOME	dGene
TOMM22	TOMM22-translocase of outer mitochondrial membrane 22 homolog (yeast)	TRANSPORTER	GO
UPF3B	UPF3B-UPF3 regulator of nonsense transcripts homolog B (yeast)	TRANSPORTER	GO
USP36	USP36-ubiquitin specific peptidase 36	PROTEASE	dGene GO
		DRUGGABLE GENOME	dGene
VDAC1	VDAC1-voltage-dependent anion channel 1	TRANSPORTER	GO
		ION CHANNEL	GO
ZC3H12D	ZC3H12D-zinc finger CCCH-type containing 12D	TUMOR SUPPRESSOR	GO

Among the Set A genes showing a change of expression influenced exclusively by the ablation of *Tis21*, there is *Pdgfd* that has been discussed in developmental (see Supplemtary Data) and migration processes as up-regulated gene. Since the overactivity of PDGF signaling can drive tumorigenesis (Pietras et al., 2003), and since PDGF-D in particular has been found to be a potent transforming and angiogenic growth factor (Li et al., 2003) highly expressed in Shh-type medulloblastoma (Yuan et al., 2013), we propose targeting PDGF-D as therapeutic strategy for medulloblastoma Shh-type, as already studied in other tumors (Heldin, 2013). Another very interesting drug target could be the phosphatidylinositol 3-kinase-related kinase Smg1, which here has been discussed in developmental and nonsense-mediated decay processes (Supplementary Material) as down-regulated in Set A (up-regulated in Set D). This protein seems to act antagonistically with mTOR signaling (González-Estévez et al., 2012; Du et al., 2014), and this is in functional synergy with the up-regulation in Set A of *Deptor*, which negatively regulates mTOR signaling (Beauchamp and Platanias, 2013). Also Deptor is one of the druggable target identified in our study. Taken together, these findings seem to support the importance of mTOR pathway and its upstream PDGF signaling in the pathogenesis of medulloblastoma (Mohan et al., 2012).

Other two druggable targets are regulators of cell cycle and developmental processes: *Sik2* and *Lats2*. The functional product of *Sik2* is localized at the centrosome where its absence leads to a delay of G1/S transition (Ahmed et al., 2010), while *Lats2* encodes for a centrosomal protein (a serin threonin kinase) whose loss leads to centrosome fragmentation (Yabuta et al., 2007), also involved in the regulation of cell cycle G1/S checkpoint, for its relevance to MB tumorigenesis due to its influence in the reduction of expression of cyclin-D1 and N-CoR (Park et al., 2012; Lit et al., 2013). The deregulation of centrosome and cilia biogenesis have been already described in different human diseases, in particular, in cancer where a derangement of cell cycle checkpoints is governed by cilia and centrosomes (Plotnikova et al., 2008; Nigg and Raff, 2009; Bettencourt-Dias et al., 2011). In addition to that, Sik2 has been characterized as negative regulator of Hippo signaling in Drosophila (Wehr et al., 2013). In our data, other two regulators of Hippo signaling appear to be down-regulated after ablation of *Tis21, Lats2*, and *Fat4* that we discuss for their role in the developmental process (Supplementary Data); both act also as tumor suppressors. This evidence supports the involvement of Hippo signaling (Roussel and Hatten, 2011) and centrosome assembly in the pathogenesis of MB.

Another putative drug target belonging to developmental processes, *Rgs5*, encodes for an endogenous repressor of Shh signaling and has been proposed in a recent study as potential therapeutic target in Hh-mediated diseases. In fact, it was shown that (i) Rgs5 inhibits the Shh-mediated signaling by activating the GTP-bound Gαi downstream of Smo and (ii) a physical complex between Rgs5 with Smo is present in primary cilia (Mahoney et al., 2013).

The apoptosis is not the only form of cellular death in which the deregulated genes in Set A are involved. In fact, the *Ripk3* functional product, a receptor-interacting protein kinase 3, has been reported to contribute to both apoptotic and necroptotic cell death, depending on target availability (Cook et al., 2014; Vanden Berghe et al., 2014). Since many anti-cancer drugs are inducers of apoptosis, the induction of RIP3-dependent necrosis is an attractive strategy to circumvent apoptosis resistance of cancer cells (Moriwaki and Chan, 2013) that is currently under investigation (Moriwaki et al., 2015). As *Ripk3* expression is induced after knockout of *Tis21* in Shh-activated background, we may hypothesize that *Ripk3* plays in our model a tumor suppressor role.

The therapeutic advantage of targeting the ubiquitin-proteasome system has already being successfully investigated with proteasomal inhibitors in Shh-type MB with *in vivo* preclinical studies (Ohshima-Hosoyama et al., 2011) and in a preliminary study with personalized targeted therapy for pediatric brain tumors among which MB (Wolff et al., 2012). However, the targeting of specific enzymes regulating the ubiquitylation process, e.g., SKP2, a SCF ubiquitin ligase, up-regulated in Set D (Figure 3), has been recently proposed as a more specific approach than the previous one (Hede et al., 2014). Two genes belonging to the ubiquitin-dependent degradation processes are up-regulated in Set A and have been identified in this study as putative drug target: *Ups36* and *Cdc27*. *Usp36*, encoding for a deubiquitinating enzyme (Quesada et al., 2004), has been detected as overexpresed in human ovarian cancer compared to normal ovaries (Li et al., 2008). An Usp36 homolog, recently detected in Drosophila stem cells, has been linked to the deubiquitylation of histone H2B and to the silencing of key differentiation genes, including genes target of Notch (Buszczak et al., 2009). Whereas the anaphase-promoting complex component Cdc27 is an E3 ubiquitin ligase able to control cell cycle progression at the G1 to S transition, and also to induce the non-proteolytic disassembly of the spindle checkpoint during mitosis (Jin et al., 2008; Manchado et al., 2010; Pawar et al., 2010). For these reasons, *Cdc27* functional product has been identified as a molecular target of the curcumin-induced cell cycle arrest and apoptosis in Shh-type MB (Lee and Langhans, 2012). The therapeutic properties of this natural substance were already shown in Shh-driven MB models, highlighting its ability to inhibit the Shh signaling, to reduce the level of β-catenin and to inhibit HDAC4 (Elamin et al., 2010; Lee et al., 2011). Thus, the up-regulation of *Cdc27* after ablation of *Tis21* may be a tumorigenic factor.

Of interest as drug target among the genes belonging to the migration processes is *Timp1*. Its signal trasduction pathway results in the migration of hNSCs, showing chemoattractant properties and being identified in the same study as a therapeutic target for human glioma (Lee et al., 2014). Here we suggest the same prospective use of Timp1 in MB Shh-type.

The *Cdc42bpb* produces a Rho GTPase activated serine/threonine kinase, which, by regulating the (non-muscle) cytoskeletal actomyosin, influences cell shape and promotes motility and migration (Tan et al., 2008, 2011), thus acting also as a key regulator of tumor cell invasion. Cdc42bpb is therefore an interesting drug target (Heikkila et al., 2011; Unbekandt et al., 2014). It's down-regulation in our Set A data is in line with the GCPs migration failure, consistently with the deregulation of others migration regulators discussed above.

Genes involved in the RNA processing are *Smg1* and *Upf3b*, respectively down- and up-regulated in Set A. Interestingly, both genes encode for proteins involved in alternative splicing (AS) coupled with Nonsense-Mediated Decay (NMD) mechanism by which NMD controls transcript abundance by regulating AS; in fact, the SURF complex, which includes the SMG1–UPF1–eRF1–eRF3 proteins, forms a bridge between the ribosome and the downstream exon-junction complex (EJC) associated with UPF3b and UPF2 (Chamieh et al., 2008; McIlwain et al., 2010). Moreover, *Smg1* has been showed to be involved in the predisposition to tumor formation and inflammation in *Smg1* heterozygous mice; this mouse model presents elevated basal tissue and serum cytokine levels—indicating low-level inflammation—and can progress to chronic inflammation or enhanced cancer development. Therefore, this is a model of inflammation-enhanced cancer development (Roberts et al., 2013), also suggesting that *Smg1* is a tumor suppressor (Du et al., 2014). To our knowledge, the possibility to target inflammation through Smg1 has never been applied to medulloblastoma until now. Furthermore, in our data *Il1b*, for example, is up-regulated in Set D and B (See Figure 2); also *Cxcl12* is up-regulated in Set D, B, and also in Set A, as mentioned previously, while *Cxcl3* is down-regulated in Set A. It is also worth noting the existence of an anticoagulant inhibitor of CXCL12/CXCR4 axis, the Tinzaparin, as well as other heparinoids that have been studied in brest cancer models for their heparinoid-mediated inhibition of chemotaxis activity (Harvey et al., 2007; Mellor et al., 2007). As Cxcl12 favors the localization of the GCPs to the EGL by chemoattraction, an inhibitor of Cxcl12 might achieve an opposite effect, similar to the promigratory effect exerted by Cxcl3.

## Summary and conclusions

During cerebellar development, Shh is expressed in Purkinje cells and regulates the proliferation of GCPs acting as mitogen (Dahmane and Ruiz i Altaba, 1999; Wallace, 1999; Wechsler-Reya and Scott, 1999). GPCs undergo a prolonged mitotic activity in the EGL during the early postnatal period in the mouse. After this intense proliferative phase, around the second postnatal week in mouse, GPCs exit the cell cycle and migrate radially to the IGL, along the Bergmann glia. During this inward migration, differentiation of post-mitotic GPCs takes place, allowing the mature granule neurons to expand the IGL, where they extend dendrites (Dyer, 2004; Rodini et al., 2010; Wang and Wechsler-Reya, 2014). As a consequence of a hyperactivation of the Shh pathway occurring in the *Ptch1*^+/−^ MB mouse model, the prolonged mitotic activity of GCPs makes them potential targets for transforming insults, leading to MB (Wang and Zoghbi, 2001).

Mutations in *Ptch1* occur in sporadic human MB and promote the tumor in *Ptch1* heterozygous mouse models at a rate of approximately 8% within 12 weeks of age and up to 30% between 12 and 25 weeks of age (Goodrich et al., 1997). As previously described (Farioli-Vecchioli et al., 2012a), the ablation of *Tis21* in our mouse model strikingly enhances, from 25 to 80%, the incidence of MB spontaneously occurring in *Ptch1* heterozygous mice. We observe that the whole balance between division, differentiation and death is disrupted in *Ptch1* heterozygous GCPs lacking *Tis21*, leading to an increase in tumor incidence (Farioli-Vecchioli et al., 2012a). In particular, Tis21 seems to act on the timing of migration of GPCs from EGL to IGL, causing an extended period of localization in the proliferative region, decrease of differentiation and increase of apoptosis (Farioli-Vecchioli et al., 2012a,b). These cellular changes are mirrored in gene expression changes occurring in Set A, shown in Table 1. We observe that the most significant enrichments of genes whose expression is altered in Set A, fall in the functional pathways of developmental signaling, retinal development, cell migration, epigenetic modulation, and primary cilium-related activities. We will summarize some consideration on these functions and also about the ubiquitin-dependent degradation (Table 1), in relation to the possible drug targets available.

### Cell cycle regulation and cell proliferation of the GCPs (Set A)

We have shown that no change in the proliferation of GCPs occurs after ablation of *Tis21* (Farioli-Vecchioli et al., 2012a). Moreover, in a recent study, we have demonstrated that the proliferation of the GCPs is not ruled by *Tis21* but by the family-related gene *Btg1* (Ceccarelli et al., 2015). Indeed, if we analyze the type of expression changes occurring in the whole array of genes of Set A that either directly or indirectly regulate the proliferation and/or the cell cycle of the GCPs (Table 7) we find that there is upregulation as well downregulation of genes that affect either positively or negatively this process, evidently resulting in no net change of proliferation of the GCPs. Nonetheless, the defect of migration of the *Tis21*-null GCPs forces them to stay a longer period in the EGL under the control of Shh influence, possibly leading to different types of alterations in cell division, including the control of centrosome assembly (see below).

**Table 7:** **The table shows a sub-set of Set A deregulated genes which can influence proliferation in a direct or indirect manner**.

**Genes**	**Functional classes**	**Expression level**	**Effect on proliferation**	**Oncosuppressor gene**
Gtpbp4	Proliferation	Up	+	
Ipo7	Cell Cycle	Up	+	
Eif3a	Cell Cycle	Up	+	
Eif3c	Cell Cycle	Up	+	
Cdc27	Cell Cycle	Up	+	
Ckap5	Cell Cycle	Up	+	
Ankrd11	Epigenetic	Up	+	
Mphosph10	Cell Cycle	Up	+	
Rps12	Proliferation	Up	+	
Rrp1	Cell Cycle	Up	+	
Srpk2	Cell Cycle	Up	+	
Taf7	Cell Cycle	Up	+	
Taok2	Cell Cycle	Up	+	
Slc6a6	Proliferation	Up	+	
Agtr2	Proliferation	Up	−	
Pa2g4	Cell Cycle	Up	−	
Eif2c1	Cell Cycle; Proliferation	Up	−	X
Pag1	Proliferation	Down	−	X
Rab11fip4	Cell Cycle	Down	−	
Lats2	Cell Cycle	Down	−	X
Tigar (9630033F20Rik)	Cell Cycle	Down	−	
Sema4b	Cell Cycle; Proliferation	Down	−	
Zc3h12d	Cell Cycle	Down	−	X
Gcnt3	Proliferation	Down	+	
Sik2	Cell Cycle	Down	+	
Wtap	Cell Cycle	Down	+	

### Cilium, GCPs migration, clathrin motility, and centrosome assembly (Set A)

We have shown a link between the Shh signaling, operating through the primary cilium, and the impairment of cell migration, i.e., the main phenotype observed in *Ptch*^+/−^*/Tis21*^*KO*^ mice [17]. In fact, the primary cilium, as mentioned above, is a sensory non-motile microtubule-based organelle which acts as a subcellular compartment for Shh signaling through a Smoothened-dependent recruitment of G_i_ proteins (Belgacem and Borodinsky, 2011). These include the Rab11 family, which impacts on cell motility, and whose components Fip4 and Fip2 are down-regulated in Set A. Remarkably, Rab11Fip2 interacts with the myosin Vb motor protein (Horgan and McCaffrey, 2009) that regulates the recycling of C-X-C chemokine receptor type 2, the receptor of Cxcl3, and the receptor-mediated chemotaxis, as confirmed by Raman et al. (2014). As we have pointed out previously, Cxcl3 induces the migration of GCPs out of the EGL and its decrease in Set A is at the origin of the increase of tumorigenesis in Tis21 KO model (Farioli-Vecchioli et al., 2012a). All this points to a link between the decrease of the Cxcl3-Cxcr2 function in Set A and the clathrin-mediated chemotaxis and microtubule-based migration. Such type of reasoning could be extended to the whole set of coiled coil molecules present in the cilium whose expression is altered in Set A, further suggesting that the ablation of *Tis21* in Set A could trigger an impairment of GCPs migration acting at more than one level.

Another interesting connection is with the cilium-based GTPase Rab11Fip4; in fact, since Rab11Fip4 induces Gli3 (Muto et al., 2007) which is a negative regulator of Shh signaling, the ablation of *Tis21*, by down-regulating *Rab11fip4*, may enhance the Shh pathway activity, thus conferring more penetrance to the Shh stimulus. Moreover, the presence of cilia is in itself necessary for the development of Shh-type MB, and the formation of cilia might be enhanced by the upregulation in Set A of *Syne2* (Chizhikov et al., 2007).

We also noticed several deregulated genes in Set A related to an evident deregulation of centrosome assembly (*Akap2, Syne2, Ckap5, Sik2, Emd*, and *Lats2*). Since the basal bodies, microtubule-based structures, are required for the formation of cilia (also non-motile ones) but also for the pericentriolar material at the core of the centrosome (Nigg and Raff, 2009), our results could confirm the previously reported evidences of a deregulation of centrosome and cilia biogenesis that have been described in different types of cancer, where a derangement of cell cycle checkpoints is governed by cilia and centrosomes (Plotnikova et al., 2008; Nigg and Raff, 2009; Bettencourt-Dias et al., 2011).

### Primary cilium (Set B)

In reference to set B (comparison of *Ptch1* heterozygous mice vs. wild-type, thus without involvement of Tis21) our attention was captured by mechanisms that could regulate cell cycle machinery in a primary cilia-dependent fashion. These are suggestive of a possible involvement of Smo-dependent non-canonical Shh-pathways, namely concerning our data showing for the first time that Plc-gamma2, Ip3r3, Trpc1, Trpc2, and Trpc3 are up-regulated in *Ptch1* heterozygous mice. These genes belong to the described Smo-dependent non-canonical Shh pathways (Figure 3) that have been reported to modulate cytoskeleton-dependent processes (Jenkins, 2009) and Ca^2+^ spikes (Brennan et al., 2012). In particular, a model in which the subcellular compartment (i.e., primary cilium) for Shh signaling allows the spatiotemporal integration of second messengers has been proposed (Belgacem and Borodinsky, 2011), and the role of Ca^2+^ signaling in granule cell turning and in modulation of their migration rate has been suggested as potential therapeutic target for some deficits in granule cell migration, since its downstream effectors control the assembly and disassembly of cytoskeletal elements (Komuro et al., 2015).

The presence of the key components of the Shh pathway in cilia has been assessed, as well as the anterograde and retrograde traffic regulating its signaling (Goetz and Anderson, 2010). We have taken in consideration the role of primary cilia in GCPs, where their presence has been assessed in the EGL at early post-natal stages (Del Cerro and Snider, 1972), as well as their requirement for Shh-induced expansion and cerebellar development (Chizhikov et al., 2007; Spassky et al., 2008). Exploring this scenario, in our MB mouse model we have highlighted some other cilia-related protein targets modified in Set B—but not in Set A—such as Tctex-1, identified as a novel “checkpoint” for G1-S transition controlling ciliary resorption, cell cycle S-phase entry and fate of neural progenitors of developing neocortex (Li et al., 2011; Sung and Li, 2011).

### Possible lineage switch of pGCPs in Set A and retinal development

An intriguing observation concerns the fact that three genes in Set A whose expression is significantly modified - namely, *Nlk, Raf1*, and *Ppp2r2b*—are markers for group 3 medulloblastoma (Kool et al., 2008; Gibson et al., 2010; Northcott et al., 2011, 2012c; Taylor et al., 2012; Hooper et al., 2014). Moreover, *Nlk* is among the genes of Set A modified in retinal development, and it has been suggested that cerebellar and retinal progenitor cells have common evolutionary origin [76]. It is also worth noting that, according to references (Kool et al., 2008; Hooper et al., 2014), among the markers for group 3 MB there are many genes involved in retinal development; in our Set A many genes as well are involved in this process, *Nlk* being common. Additionally, in Set A there are at least two genes whose expression is modified, *Gli1* and *Pdgfd*, which are markers of Shh-type medulloblastoma (Kool et al., 2008; Gibson et al., 2010; Northcott et al., 2011, 2012c; Taylor et al., 2012; Hooper et al., 2014). Hence, the ablation of Tis21 causes changes in the *Ptch1* heterozygous Shh-type model of two Shh-type MB marker genes (increased expression) and of three group 3 MB marker genes (Table 1). As a whole, these data may suggest the possibility that the ablation of *Tis21*, by altering the expression of important Shh marker genes such *Pdgfd* and *Gli1*, may increase the penetrance of the Shh-type tumor phenotype, but also the possibility of a shift of the Shh phenotype toward the group 3 MB. A possible shift toward group 3, associated with retinal development control, may underlie the intriguing novel concept that the inactivation of a gene—in this case *Tis21*, which is known to be required for the terminal differentiation of neural stem cells (Micheli et al., 2015)—may favor in Shh-activated GCPs a lineage shift toward other neural cell types involved in group 3 MB onset. Further analyses will be necessary to clarify this possibility.

A further correlation concerns the up-regulation of *Deptor* in Set A: this gene has been remarkably associated with reduced differentiation and increase of regenerative potential of pluripotent stem cells (Agrawal et al., 2014). *Deptor* functional product also inhibits the TOR pathway, whose activation results in a more penetrant phenotype in Shh-type MB, with enhanced survival of cancer stem cells (Beauchamp and Platanias, 2013). Thus, we can further suppose that the ablation of *Tis21* enhances the stem cell character of the Shh–activated GCPs as a preliminary step to possible lineage shifts.

### Epigenetic changes in GCPs of Set A

The most significant enrichment in Set A is probably observed for genes that regulate transcription epigenetically. We have previously performed a functional genomic analysis where we identified more than 30 genes altered by the *Tis21* KO genotype relative to *Tis21* wild-type, (in background either *Ptch1* wild-type or heterozygous) and involved in epigenetic control, being regulated by DNA methylation or histone deacetylation, or being able to associate with HDAC1 or HDAC4 (Farioli-Vecchioli et al., 2012b). We limited the present analysis within Set A to genes acting as histone modifier and their regulators or involved in chromatin remodeling, finding several of the first class and one of the second. Among them, is relevant *PadI4*, which by demethylating histones may act as a tumor suppressor (Tanikawa et al., 2012); thus, its down-regulation in Set A could enhance tumorigenesis. Remarkable is also the series of histone modification editors ANKRDs, whose genes are down-regulated in SetA. Among the chromatin modifiers, we find *Dek*, up-regulated in Set A and also up-regulated in group 4 MB (Hooper et al., 2014), which is a known oncogene that can confer stem cell-like qualities and is thus potentially enhancing the probability of cancer (Privette Vinnedge et al., 2013). Altogether, the alteration in Set A of genes involved in histone modification and chromatin remodeling fits with the idea that the ablation of *Tis21* may reduce in the *Tis21*-null GCPs the restraint toward a lineage shift, as exposed in the previous section.

### RNA processing in Set A

The role of *Tis21* in the cell proliferation control has been recently associated in human cells to RNA deadenylation, by which it influences mRNA poly(A) tail shortening (Stupfler et al., 2016). Nevertheless, in Set A we detect the deregulation of genes related to AS or to NMD, but not to mRNA deadenylation.

### Drug targets

This is a very interesting topic for therapy, that will need preclinical experimental studies for evaluation. Among the Set A genes that could be targeted by a drug, are worth mentioning: (i) the inhibitor of Pdgfd (ligand for Pdgfrb), since the activation of the PDGFR pathway has been shown to activate the Cxcl12 receptor; (ii) the possibility to enhance the activity of the tumor suppressor phosphatidylinositol 3-kinase-related kinase Smg1, whose ablation favors inflammation and cancer development. This could also be obtained by negatively targeting mTOR, which is antagonistic to Smg1 (possibly also by enhancing Deptor activity, which negatively regulates mTOR signaling). Moreover, as mentioned above, the intracerebellar administration of Cxcl3 functional product, by controlling the timing of migration of pre-neoplastic pGCPS, may have therapeutic effects that still need to be fully tested *in vivo*. Since these genes are deregulated in a Shh-type MB whose frequency is enhanced by *Tis21* ablation, and since Tis21 has been shown to be down-regulated in human MBs (mainly Shh-type), it is plausible a benefit using Cxcl3 in MB therapy of at least the Shh-type.

## Author contributions

GG analyzed the data; SC, FT designed the experiments; GG, FT wrote the paper; MC, LM carried out experimental work; GG, FT, and SC are responsible for accuracy and integrity of any part of the work.

## Funding

This work was supported by grants from the Italian Ministry of Economy and Finance (Project FaReBio) to FT, the CNR Project DSB.AD004.094 to FT, and by the Italian Ministry of Education, Universities and Research grant CTN01_00177_817708 to SC. MC is recipient of fellowships from the Italian Foundation for Cancer Research (FIRC; year 2014) and from Fondazione Santa Lucia (year 2015), while GG is recipient of the fellowship from the Italian Ministry of Education, Universities and Research grant CTN01_00177_817708 (2014–2015).

### Conflict of interest statement

The authors declare that the research was conducted in the absence of any commercial or financial relationships that could be construed as a potential conflict of interest. However, a patent was filed by the National Research Council on the possible use of the chemokine Cxcl3 in medulloblastoma therapy. No financial exploitation of the patent has occurred.
