# Supplementary material for: Functional Genomics Identifies Tis21-Dependent Mechanisms and Putative Cancer Drug Targets Underlying Medulloblastoma Shh-Type Development
Source: Front Pharmacol. 2016 Nov 30;7:449. doi: 10.3389/fphar.2016.00449 (PMC5127835; doi:10.3389/fphar.2016.00449)
Supplement: Supplementary file 1 [file DataSheet1.pdf]

Supplementary Material

FUNCTIONAL GENOMICS IDENTIFIES TIS21-DEPENDENT MECHANISMS AND PUTATIVE CANCER DRUG TARGETS UNDERLYING MEDULLOBLASTOMA SHH-TYPE DEVELOPMENT

Giulia Gentile[1], Manuela Ceccarelli[2], Laura Micheli[2], Felice Tirone[2], Sebastiano Cavallaro[1]

[1] Institute of Neurological Sciences, National Research Council, Catania, Italy;

[2] Institute of Cell Biology and Neurobiology, National Research Council, Fondazione Santa Lucia, Rome, Italy.

Correspondence:
Felice Tirone email: felice.tirone@cnr.it;

Sebastiano Cavallaro email:sebastiano.cavallaro@cnr.it

1 Supplementary Data

1.1 Early post-natal development

A complex signaling cross-talk between different developmental cascades, which if deregulated, acquire oncogenic effects and the GCPs are the targets of tumor transformation in the MB Shh-driven (Mimeault and Batra, 2011; Manoranjan et al., 2012). Our analysis revealed numerous deregulated genes belonging to the developmental pathways, either down-regulated (i.e. ***Gpr82***, ***Smg1***, ***H19***, ***Mettl14***, ***Fat4***, ***Sema4b***, ***Lats2***) or up-regulated (i.e. ***Rgs5***, ***Sgsm2***, ***Emd***, ***Rab18*****,** ***Vps35***, ***Nlk***, ***Gigyf2***, ***Kctd5***, ***Ankrd11*****,** ***Cxcl12*** and ***Pdgfd***).

The protein encoded by the ***Gpr82*** is an orphan G protein-coupled receptor of unknown function (Lee et al., 2001). ***Smg1*** is known to be required for embryogenesis since using a gene-trap model of *Smg1* deficiency it has been showed that its loss is lethal at embryonic day 8.5 (McIlwain et al., 2010). ***H19*** is a gene encoding for an imprinted maternally expressed transcript (non-protein coding), located downstream the growth-promoting insulin-like growth factor 2 (Igf2), with which shares a common imprinting mechanism; in fact, their variable imprinting has been observed in fetal cerebellum and MB (Albrecht et al., 1996). Growth in the developing mouse embryo is largely governed by Igf2 and Shh can transcriptionally activate *Igf2* (Chao and D'Amore, 2008). *H19* has been reported both as oncogene and tumor suppressor but also in fetal growth syndromes in humans (Guo et al., 2014; Matouk et al., 2014; Park et al., 2014). *H19* is also a microRNA precursor whose expression results in the post-transcriptional down-regulation of specific mRNAs during vertebrate development (Cai and Cullen, 2007) and inhibits cell proliferation (Keniry et al., 2012) (*H19* is down-regulated in Set A). Furthermore, H19 has been shown in mice to act as trans-regulator of a group of co-expressed genes belonging to the imprinted gene network controlling fetal and early postnatal growth (Gabory et al., 2010). The ***Mettl14*** gene is instead a modulator of RNA stability in embryonic stem cells, since through its activity of RNA methylation (epitranscriptomics) plays an important role in RNA processing and metabolism, by destabilizing the mRNAs encoding of developmental regulators (m^6A methylation is inversely correlated with mRNA stability and gene

expression) (Lin and Gregory, 2014; Wang et al., 2014). Concerning the gene **Fat4**, a decrease of its expression in the mouse embryonic neuroepithelium has been correlated with an increase of the number of cortical progenitor cells and decrease of their differentiation into neurons; such effect is counteracted by the activation of the Hippo signaling pathway, which implicates Fat4 as key regulator of the mammalian neurogenesis (Cappello et al., 2013). ***Sema4b*** is mainly expressed in glial cells of the developing cerebellum (Maier et al., 2011). Semaphorin-plexin signaling is activated from the binding of Semaphorin-4B to Plexin-B2 (Humbert and Godde, 2015), an event which in non-small lung cancer cells seems to promote the tumor invasion (Jian et al., 2014a; Jian et al., 2014b). The Serine/threonine-protein kinase **Lats2**, by negatively regulating YAP1 in the Hippo signaling pathway, known to act in MBs in concert with the Shh pathway (Roussel and Hatten, 2011), prevents DNA damage-induced apoptosis (Reuven et al., 2013) and controls the TGFβ-SMAD pathway (Varelas et al., 2010).

Among the up-regulated genes in Set A, the ***Rgs5*** encodes for an endogenous repressor of Shh signaling whose therapeutic role has been discussed in the main text (paragraph 2.2.8). and has been proposed in a recent study as potential therapeutic target in Hh-mediated diseases. In fact, it was shown that i) Rgs5 inhibits the Shh-mediated signaling by activating the GTP-bound Gαi downstream of Smo and ii) a physical complex between Rgs5 with Smo is present in primary cilia (Mahoney et al., 2013). *Sgsm2* product functions as a modulator of the RAP and RAB subfamily members of the vesicle transportation small G protein superfamily (Yang et al., 2007). ***Emd*** gene encodes for the Emerin protein that has been proposed to block or attenuate the nuclear accumulation of at least three signaling proteins: ERK1/2, Lmo7 and β-catenin (Berk et al., 2013). Concerning *Rab18*, interestingly, loss-of-function mutations of this gene have been found to cause the Warburg Micro syndrome which is associated with cerebellar and cerebellar vermis hypoplasia; moreover, it is also known a correlation in human healthy adults of the *Rab18* gene polymorphism (rs3765133) with cerebellar development and in particular with its volume (Bem et al., 2011; Cheng et al., 2014). The ***Vps35*** gene product is known to regulate the Wnt signaling through its cargo activity, since the loss of Vps35 function prevents the endosome-to-Golgi recycling of Wntless, a protein essential for secretion of Wnt ligands (Berwick and Harvey, 2014). *Nlk* encodes for a negative regulator of Wnt/β-Catenin signaling, which is activated by the non-canonical Wnt-5a/Ca^{2+} pathway (Ishitani et al., 2003) This latter has been described as involved in the pathogenesis of MB group C, or 3 (Northcott et al., 2011; Chen et al., 2013) and, remarkably, Wnt-5a is heavily down-regulated by the ablation of *Tis21* (Set A) but is up-regulated in conditions of heterozygosity of *Ptch1* (in Set B and D). This fact may imply that the ablation of *Tis21* increases MB tumorigenesis by modulating genes implied in group 3 tumors. Other genes modified in Set A (although not significantly) and belonging to group 3 are *Ppp2r2b* and *Raf1* ((Kool et al., 2008; Gibson et al., 2010; Northcott et al., 2011; Northcott et al., 2012; Taylor et al., 2012; Hooper et al., 2014) see Conclusions). Concerning the ***Gigyf2*** gene, its protein interacts with the GRB10 adapter that in turn modulates the IGF-I receptor signaling (Giovannone et al., 2003); *Gigyf2* is also deregulated in Set B and Set D (see fig.3). The K-potassium channel tetramerization domain protein encoded by ***Kctd5*** has been identified as a substrate-specific adaptor for cullin3-based E3 ligases (Bayon et al., 2008; Balasco et al., 2014), whose Cul3-mediated ubiquitination has different actions. Namely, the control of different cell-cycle phases (Singer et al., 1999; Sumara et al., 2007; Maerki et al., 2009; Beck et al., 2013), the regulation of intracellular trafficking, in particular secretion and endosome maturation (Hubner and Peter, 2012; Huotari et al., 2012). Moreover, Cul3-mediated ubiquitination is involved in ubiquitination and proteasomal degradation of different proteins, including GLI2 and GLI3 in complex with the substrate-binding adaptor Spop (Wang et al., 2010). As mentioned in the main text, many proteins belonging to the ubiquitin-dependent degradation within the GCPs are deregulated in Set B and Set D, meaning that

the ablation of *Tis21* has different effects whether it occurs in a wildtype background or heterozygous for *Ptch1* (see fig.3). The functional product of ***Ankrd11*** is a chromatin regulator controlling histone acetylation and gene expression during neural development; in fact, knockdown of *Ankrd11* in developing murine or human cortical neural precursors has been shown to cause decreased proliferation, reduced neurogenesis and aberrant neuronal positioning (Gallagher et al., 2015). The chemokine Cxcl12, encoded by the ***Cxcl12*** gene, is known to play a central role in normal cerebellar development by influencing both the migration and proliferation of cerebellar granule cells (Klein et al., 2001), and consequently it plays an important role in MB pathogenesis (Ozawa et al., 2014). In particular, a new molecular subgroup of MB characterized by the coactivation of the SHH and CXCL12/CXCR4 pathways has been identified in human youngest patients in association with desmoplastic histology (Sengupta et al., 2012). This is of particular relevance if we consider that *Cxcl12* expression is heavily increased by the ablation of *Tis21* in GCPs. Finally, the Pdgfd gene encodes for a protein known to have an important role in the regulation of physiological and pathological cell growth (LaRochelle et al., 2002; Heldin, 2013); its function in MB migration together with the CXCL12/CXCR4 signaling has been discussed in the section treating the migration of the GCPs (Yuan et al., 2013).

1.2 Epigenetic modulation.

Hist2h2bb (down-regulated in Set A) and ***Hist3h2ba*** (up-regulated in Set A) are described as pseudogenes in human but have their histone functional products in mouse (Marzluff et al., 2002; Gonzalez-Romero et al., 2010) and are known as replication-dependent histone genes (Marzluff et al., 2002).

Among the *down-regulated* genes in Set A, we also detected the ***Cbx3*** gene, which encodes for the Heterochromatin protein 1. This protein has been primarily identified as a reader, able to recognize and bind methylated histone H3 at Lys9, leading to the epigenetic repression of differentiation (Arney and Fisher, 2004). Moreover, it has been shown to be responsible for the histone H4 K20 trimethylation, through which epigenetically controls both cell differentiation and cancer development, suggesting its importance as cancer therapeutic target (Takanashi et al., 2009). ***Padi4*** (peptidylarginine deiminase 4) encodes for a protein that mediates gene expression by demethylating histones, i.e., by converting methyl-Arg residues of histones H3, H4 as well as H1 to citrulline and releasing methylamine (Wang et al., 2004). Notably, through H1 citrullination, *Padi4* activates pluripotency of pluripotent cells in the early mouse embryo, since citrullination of a single arginine residue within the DNA-binding site of H1 results in its displacement from chromatin and global chromatin decondensation (Christophorou et al., 2014). Recently, this protein has shown to citrullinate also the DNA (cytosine-5)-methyltransferase 3A, regulating its DNA methyltransferases activity (Deplus et al., 2014). Compared to benign and non-tumor diseases, many malignant tumor types exhibit increased peptidylarginine deiminase 4 levels in tumorous cells, highlighting its importance in the promotion of tumorigenesis (Chang et al., 2009). In particular, it has been shown to regulate tumor suppressor gene expression acting as a corepressor of p53 to regulate SESN2-mTORC1 autophagy pathway (Wang et al., 2012). Interestingly, the citrullination of H4R3 and Lamin C has been negatively correlated with p53 protein expression and with tumor size in non-small cell lung cancer tissues, suggesting that peptidylarginine deiminase 4 could function as a tumor suppressor that mediates the apoptotic process of damaged cells (Tanikawa et al., 2012). In analogy, the decrease of *Padi4* observed in SetA, might be associated with the enhancement of tumorigenicity occurring in *Tis21*-null GCPs.

Among the *up-regulated* genes in Set A, we detected three genes belonging to the histone modification editors ANKRDs (Plass et al., 2013), i.e. ***Ankrd11***, ***Ankrd24*** and ***Ankrd26***. *Ankrd11*

has been reported as MB antigen (Behrends et al., 2003), is a recruiter of histone deacetylases to the p160 coactivators/nuclear receptor complex to inhibit ligand-dependent transactivation (Zhang et al., 2004) and regulates proliferation and neurogenesis in the embryonic brain (Gallagher et al., 2015), while *Ankrd26* has been linked to the glucose homeostasis (Raciti et al., 2011). ***Brwd1*** encodes a transcriptional activator containing bromodomains by which binds histone acetyl groups, thus has been classified in the histone modification readers (Arrowsmith et al., 2012; Filippakopoulos and Knapp, 2012; Plass et al., 2013). This nuclear protein is broadly expressed in the mouse embryo and has been associated with a SWI/SNF chromatin remodeling complex component (Huang et al., 2003). Recently, it has been identified as human putative motility modifier, involved in cell morphology and cytoskeleton organization (Bai et al., 2011). ***Dek*** is known to be an oncogene, up-regulated in group 4 MB (Hooper et al., 2014). Its functional product functions as an ''architectural'' protein in chromatin (Cavellan et al., 2006; Hu et al., 2007), which can be shuttled from the extracellular space to the intracellular (Saha et al., 2013); remarkably, in agreement with its increase observed in Set A, *Dek functional product* can confer stem cell-like qualities, thus potentially leading to cancer (Privette Vinnedge et al., 2013).

The **histone modifier regulators** up-regulated in our Set A data were *Anp32a*, *Taf7*, *Pag2g4, Ipo7* and *Emd*. ***Anp32a*** encodes for a member of the INHAT (inhibitor of histone acetyltransferase) complex that binds to histones and masks accessibility of lysines of histone tails (Seo et al., 2001), and whose depletion promotes neurite outgrowth *in vivo*, likely by regulating the expression of the *Nf-L* (a neuron-specific cytoskeletal gene), through binding to its promoter and modulating histone acetylation levels (Kular et al., 2009). Thus, the increase of *Anp32a* expression would be in line with the decreased differentiation observed in *Tis21*–null GCPs. This gene has been also described as a tumor suppressor, repressing cell growth through the inhibition of transcription, thanks to its ability to block acetylation and phosphorylation of histone H3 and to initiate its proapoptotic activity (Fan et al., 2006). The ***Taf7*** gene product is a subunit of TFIID, known to inhibit the acetyltransferase activity of the Transcription initiation factor TFIID subunit (Gegonne et al., 2001); in this way *Taf7* functional product acts as a transcriptional repressor of the expression of Cyclin D1 and Cyclin A genes, thus acting as cell cycle regulator of G1/S phase (Kloet et al., 2012; Gegonne et al., 2013). Moreover, this protein regulates transcription of both TAF1-dependent and -independent genes, emerging as a critical regulator of transcription initiation and cell proliferation (Gegonne et al., 2013). However, we do not find consistency in the expected action of *Taf7* since its large increase in Set A is not matched by a corresponding decrease of *cyclin D1* levels, suggesting that further interactions exist. *Pa2g4* gene protein, interestingly, represses transcription of some E2F-regulated promoters via its ability to recruit HDAC activity (Zhang et al., 2003). Finally, Emerin (*Emd*) protein is known to be associated with the core components of the N-CoR complex and to bind directly Histone deacetylase 3 (Berk et al., 2013); in this complex, the functional product of ***Ipo7*** is known to mediate the nuclear import of H1 histone and the core histones H2A, H2B, H3 and H4 (Jakel et al., 1999; Muhlhausser et al., 2001).

1.3 RNA Processing and Nonsense-Mediated Decay mechanisms; Ribosome-related mechanisms.

During the data analysis we also noticed an interesting involvement of deregulated genes of Set A that act as regulators or targets of RNA processing as well as of Nonsense-Mediated Decay (NMD) mechanism, or are involved in translation initiation and ribosome-related mechanisms (i.e. biogenesis, processing and transport). In particular, there are two targets of alternative splicing (AS) among the genes discussed in the main text, i.e., ***Rab11fip4***, whose proteic product regulates

receptor-mediated endocytosis coupled with cytoskeletal remodeling and microtubule-based vesicle trafficking, and ***Ehbp1*** that has been reported among those affected by alternative splicing in Shh-associated MB (Menghi et al., 2011)**.**

The importance of **AS patterns** into the genetic determination of diseases, among which cancer, is currently under study but it has already revealed new insights (Xiong et al., 2015). In consideration of this evidence, we have detected the deregulation of many genes of Set A that are involved in AS: i) an AS regulator of apoptotic genes (Bonnal et al., 2008; Fushimi et al., 2008) and of NUMB protein through which affects cancer cell proliferation (Bechara et al., 2013), i.e. the RNA-binding protein 5 encoded by ***Rbm5*** tumor suppressor gene (Mourtada-Maarabouni et al., 2006); ii) the pre-mRNA-splicing regulator ***WTAP*** (Ortega et al., 2003) that has been shown to interact with the functional product of ***Mettl14*** as a regulatory subunit of the m6A methyltransferase complex playing a critical role in epitranscriptomic regulation of RNA metabolism and RNA splicing (Liu et al., 2014; Ping et al., 2014); iii) a target of splicing, i.e. ***Ehbp1***, previously demonstrated to undergo SHH-associated splicing (Menghi et al., 2011) and involved in clathrin-mediated endocytosis linked to cytoskeleton actin reorganization (Guilherme et al., 2004); iv) the ***Rab11fip4*** transcript A (Muto et al., 2007) as well as another target of AS, i.e., v) the RNA-binding protein ***RALY*** (Khrebtukova et al., 1999), homolog of a human heterogeneous nuclear ribonucleoprotein that showed to have pleiotropic effects on RNA metabolism and translation (Tenzer et al., 2013); vi) the ***Srpk2*** functional product, , the SRSF protein kinase 2, required for spliceosomal B complex formation and the phosphorylation of the protein DDX23, encoded by ***Ddx23*** gene (up-regulated in Set A) (Mathew et al., 2008); furthermore, ***Srpk2*** interacts with the component of the splicing-dependent multiprotein exon junction complex Acinus in the regulation of Leukemia tumorigenesis (Jang et al., 2008); vii) the splicing associated factor ***Dek*** (Le Hir et al., 2000); Viii) the transcription-splicing factor ***Htatsf1*** that regulates, among the others, genes involved in the cell cycle (Miller et al., 2011).

We have also detected the deregulation of two genes involved in the AS-coupled NMD mechanism, i.e. ***Smg1*** and ***Upf3b*** that are respectively down and up-regulated in Set A. Their role has been discussed more in detail in the main text (paragraph 2.2.8).

Other evidences in our data reveal a deregulation of the ***translation activities and other ribosome-related mechanisms***. Three translation initiation factors are up-regulated in Set A, i.e. ***Eif2c1***, ***Eif3a*** and ***Eif3c***, known for their role in increased protein synthesis supporting tumor development (Parisi et al., 2011; Hershey, 2014). ***Rmnd1*** functional product is known for its role in mitochondrial translation, possibly by coordinating the assembly or maintenance of the mitochondrial ribosome (Janer et al., 2012). Concerning the others ribosome-related mechanisms, a certain number of deregulated genes in Set A are involved in ribosome biogenesis (i.e. ***Rrp1*** (Yoshikawa et al., 2011), ***Gtpbp4*** (Lapik et al., 2007)), a 40S ribosomal component (i.e. ***Rps12***), a pre-18S ribosomal RNA processing (i.e. ***Mphosph10***) (Granneman et al., 2003) and a nuclear import of ribosomal proteins (i.e. encoded by ***Ipo7*** (Jakel and Gorlich, 1998)). Notably, the overexpression of genes involved in ribosomal functions, such as *Rps20* and *Rpl30* that encode for a component of the 60S ribosomal subunit 40S subunit respectively, has been already associated with adverse outcome in medulloblastoma (De Bortoli et al., 2006).

Albrecht, S., Waha, A., Koch, A., Kraus, J.A., Goodyer, C.G., and Pietsch, T. (1996). Variable imprinting of H19 and IGF2 in fetal cerebellum and medulloblastoma. *J Neuropathol Exp Neurol* 55(12)**,** 1270-1276.

Arney, K.L., and Fisher, A.G. (2004). Epigenetic aspects of differentiation. *J Cell Sci* 117(Pt 19)**,** 4355-4363. doi: 10.1242/jcs.01390.

Arrowsmith, C.H., Bountra, C., Fish, P.V., Lee, K., and Schapira, M. (2012). Epigenetic protein families: a new frontier for drug discovery. *Nat Rev Drug Discov* 11(5)**,** 384-400. doi: 10.1038/nrd3674.

Bai, S.W., Herrera-Abreu, M.T., Rohn, J.L., Racine, V., Tajadura, V., Suryavanshi, N., et al. (2011). Identification and characterization of a set of conserved and new regulators of cytoskeletal organization, cell morphology and migration. *BMC Biol* 9**,** 54. doi: 10.1186/1741-7007-9-54.

Balasco, N., Pirone, L., Smaldone, G., Di Gaetano, S., Esposito, L., Pedone, E.M., et al. (2014). Molecular recognition of Cullin3 by KCTDs: insights from experimental and computational investigations. *Biochim Biophys Acta* 1844(7)**,** 1289-1298. doi: 10.1016/j.bbapap.2014.04.006.

Bayon, Y., Trinidad, A.G., de la Puerta, M.L., Del Carmen Rodriguez, M., Bogetz, J., Rojas, A., et al. (2008). KCTD5, a putative substrate adaptor for cullin3 ubiquitin ligases. *FEBS J* 275(15)**,** 3900-3910. doi: 10.1111/j.1742-4658.2008.06537.x.

Bechara, E.G., Sebestyen, E., Bernardis, I., Eyras, E., and Valcarcel, J. (2013). RBM5, 6, and 10 differentially regulate NUMB alternative splicing to control cancer cell proliferation. *Mol Cell* 52(5)**,** 720-733. doi: 10.1016/j.molcel.2013.11.010.

Beck, J., Maerki, S., Posch, M., Metzger, T., Persaud, A., Scheel, H., et al. (2013). Ubiquitylation-dependent localization of PLK1 in mitosis. *Nat Cell Biol* 15(4)**,** 430-439. doi: 10.1038/ncb2695.

Behrends, U., Schneider, I., Rossler, S., Frauenknecht, H., Golbeck, A., Lechner, B., et al. (2003). Novel tumor antigens identified by autologous antibody screening of childhood medulloblastoma cDNA libraries. *Int J Cancer* 106(2)**,** 244-251. doi: 10.1002/ijc.11208.

Bem, D., Yoshimura, S., Nunes-Bastos, R., Bond, F.C., Kurian, M.A., Rahman, F., et al. (2011). Loss-of-function mutations in RAB18 cause Warburg micro syndrome. *Am J Hum Genet* 88(4)**,** 499-507. doi: 10.1016/j.ajhg.2011.03.012.

Berk, J.M., Tifft, K.E., and Wilson, K.L. (2013). The nuclear envelope LEM-domain protein emerin. *Nucleus* 4(4)**,** 298-314. doi: 10.4161/nucl.25751.

Berwick, D.C., and Harvey, K. (2014). The regulation and deregulation of Wnt signaling by PARK genes in health and disease. *J Mol Cell Biol* 6(1)**,** 3-12. doi: 10.1093/jmcb/mjt037.

Bonnal, S., Martinez, C., Forch, P., Bachi, A., Wilm, M., and Valcarcel, J. (2008). RBM5/Luca-15/H37 regulates Fas alternative splice site pairing after exon definition. *Mol Cell* 32(1)**,** 81-95. doi: 10.1016/j.molcel.2008.08.008.

Cai, X., and Cullen, B.R. (2007). The imprinted H19 noncoding RNA is a primary microRNA precursor. *RNA* 13(3)**,** 313-316. doi: 10.1261/rna.351707.

Cappello, S., Gray, M.J., Badouel, C., Lange, S., Einsiedler, M., Srour, M., et al. (2013). Mutations in genes encoding the cadherin receptor-ligand pair DCHS1 and FAT4 disrupt cerebral cortical development. *Nat Genet* 45(11)**,** 1300-1308. doi: 10.1038/ng.2765.

Cavellan, E., Asp, P., Percipalle, P., and Farrants, A.K. (2006). The WSTF-SNF2h chromatin remodeling complex interacts with several nuclear proteins in transcription. *J Biol Chem* 281(24)**,** 16264-16271. doi: 10.1074/jbc.M600233200.

Chang, X., Han, J., Pang, L., Zhao, Y., Yang, Y., and Shen, Z. (2009). Increased PADI4 expression in blood and tissues of patients with malignant tumors. *BMC Cancer* 9**,** 40.

Chao, W., and D'Amore, P.A. (2008). IGF2: epigenetic regulation and role in development and disease. *Cytokine Growth Factor Rev* 19(2)**,** 111-120. doi: 10.1016/j.cytogfr.2008.01.005.

Chen, P.K., Fan, Y.B., Man, T.K., Hung, Y., Lau, C.C., and Wong, S.T.C. (2013). A gene signature based method for identifying subtypes and subtype-specific drivers in cancer with an application to medulloblastoma. *Bmc Bioinformatics* 14. doi: Artn S1

Doi 10.1186/1471-2105-14-S18-S1.

Cheng, C.Y., Yang, A.C., Huang, C.C., Liu, M.E., Liou, Y.J., Wu, J.C., et al. (2014). The association of RAB18 gene polymorphism (rs3765133) with cerebellar volume in healthy adults. *Cerebellum* 13(5)**,** 616-622. doi: 10.1007/s12311-014-0579-y.

Christophorou, M.A., Castelo-Branco, G., Halley-Stott, R.P., Oliveira, C.S., Loos, R., Radzisheuskaya, A., et al. (2014). Citrullination regulates pluripotency and histone H1 binding to chromatin. *Nature* 507(7490)**,** 104-108. doi: 10.1038/nature12942.

De Bortoli, M., Castellino, R.C., Lu, X.Y., Deyo, J., Sturla, L.M., Adesina, A.M., et al. (2006). Medulloblastoma outcome is adversely associated with overexpression of EEF1D, RPL30, and RPS20 on the long arm of chromosome 8. *BMC Cancer* 6**,** 223. doi: 10.1186/1471-2407-6-223.

Deplus, R., Denis, H., Putmans, P., Calonne, E., Fourrez, M., Yamamoto, K., et al. (2014). Citrullination of DNMT3A by PADI4 regulates its stability and controls DNA methylation. *Nucleic Acids Res* 42(13)**,** 8285-8296. doi: 10.1093/nar/gku522.

Fan, Z., Zhang, H., and Zhang, Q. (2006). Tumor suppressor pp32 represses cell growth through inhibition of transcription by blocking acetylation and phosphorylation of histone H3 and initiating its proapoptotic activity. *Cell Death Differ* 13(9)**,** 1485-1494. doi: 10.1038/sj.cdd.4401825.

Filippakopoulos, P., and Knapp, S. (2012). The bromodomain interaction module. *FEBS Lett* 586(17)**,** 2692-2704. doi: 10.1016/j.febslet.2012.04.045.

Fushimi, K., Ray, P., Kar, A., Wang, L., Sutherland, L.C., and Wu, J.Y. (2008). Up-regulation of the proapoptotic caspase 2 splicing isoform by a candidate tumor suppressor, RBM5. *Proc Natl Acad Sci U S A* 105(41)**,** 15708-15713. doi: 10.1073/pnas.0805569105.

Gabory, A., Jammes, H., and Dandolo, L. (2010). The H19 locus: role of an imprinted non-coding RNA in growth and development. *Bioessays* 32(6)**,** 473-480. doi: 10.1002/bies.200900170.

Gallagher, D., Voronova, A., Zander, M.A., Cancino, G.I., Bramall, A., Krause, M.P., et al. (2015). Ankrd11 is a chromatin regulator involved in autism that is essential for neural development. *Dev Cell* 32(1)**,** 31-42. doi: 10.1016/j.devcel.2014.11.031.

Gegonne, A., Devaiah, B.N., and Singer, D.S. (2013). TAF7: traffic controller in transcription initiation. *Transcription* 4(1)**,** 29-33. doi: 10.4161/trns.22842.

Gegonne, A., Weissman, J.D., and Singer, D.S. (2001). TAFII55 binding to TAFII250 inhibits its acetyltransferase activity. *Proc Natl Acad Sci U S A* 98(22)**,** 12432-12437. doi: 10.1073/pnas.211444798.

Gibson, P., Tong, Y., Robinson, G., Thompson, M.C., Currle, D.S., Eden, C., et al. (2010). Subtypes of medulloblastoma have distinct developmental origins. *Nature* 468(7327)**,** 1095-1099. doi: 10.1038/nature09587.

Giovannone, B., Lee, E., Laviola, L., Giorgino, F., Cleveland, K.A., and Smith, R.J. (2003). Two novel proteins that are linked to insulin-like growth factor (IGF-I) receptors by the Grb10 adapter and modulate IGF-I signaling. *J Biol Chem* 278(34)**,** 31564-31573. doi: 10.1074/jbc.M211572200.

Gonzalez-Romero, R., Rivera-Casas, C., Ausio, J., Mendez, J., and Eirin-Lopez, J.M. (2010). Birth-and-death long-term evolution promotes histone H2B variant diversification in the male germinal cell line. *Mol Biol Evol* 27(8)**,** 1802-1812. doi: 10.1093/molbev/msq058.

Granneman, S., Gallagher, J.E., Vogelzangs, J., Horstman, W., van Venrooij, W.J., Baserga, S.J., et al. (2003). The human Imp3 and Imp4 proteins form a ternary complex with hMpp10, which only interacts with the U3 snoRNA in 60-80S ribonucleoprotein complexes. *Nucleic Acids Res* 31(7)**,** 1877-1887.

Guilherme, A., Soriano, N.A., Bose, S., Holik, J., Bose, A., Pomerleau, D.P., et al. (2004). EHD2 and the novel EH domain binding protein EHBP1 couple endocytosis to the actin cytoskeleton. *J Biol Chem* 279(11)**,** 10593-10605. doi: 10.1074/jbc.M307702200.

Guo, G., Kang, Q., Chen, Q., Chen, Z., Wang, J., Tan, L., et al. (2014). High expression of long non-coding RNA H19 is required for efficient tumorigenesis induced by Bcr-Abl oncogene. *FEBS Lett* 588(9)**,** 1780-1786. doi: 10.1016/j.febslet.2014.03.038.

Heldin, C.H. (2013). Targeting the PDGF signaling pathway in tumor treatment. *Cell Commun Signal* 11**,** 97. doi: 10.1186/1478-811X-11-97.

Hershey, J.W. (2014). The role of eIF3 and its individual subunits in cancer. *Biochim Biophys Acta*. doi: 10.1016/j.bbagrm.2014.10.005.

Hooper, C.M., Hawes, S.M., Kees, U.R., Gottardo, N.G., and Dallas, P.B. (2014). Gene expression analyses of the spatio-temporal relationships of human medulloblastoma subgroups during early human neurogenesis. *PLoS One* 9(11)**,** e112909. doi: 10.1371/journal.pone.0112909.

Hu, H.G., Scholten, I., Gruss, C., and Knippers, R. (2007). The distribution of the DEK protein in mammalian chromatin. *Biochem Biophys Res Commun* 358(4)**,** 1008-1014. doi: 10.1016/j.bbrc.2007.05.019.

Huang, H., Rambaldi, I., Daniels, E., and Featherstone, M. (2003). Expression of the Wdr9 gene and protein products during mouse development. *Dev Dyn* 227(4)**,** 608-614. doi: 10.1002/dvdy.10344.

Hubner, M., and Peter, M. (2012). Cullin-3 and the endocytic system: New functions of ubiquitination for endosome maturation. *Cell Logist* 2(3)**,** 166-168. doi: 10.4161/cl.20372.

Humbert, P.O., and Godde, N.J. (2015). Mitotic spindle orientation: semaphorin-plexin signaling flags the way. *Dev Cell* 33(3)**,** 243-244. doi: 10.1016/j.devcel.2015.04.012.

Huotari, J., Meyer-Schaller, N., Hubner, M., Stauffer, S., Katheder, N., Horvath, P., et al. (2012). Cullin-3 regulates late endosome maturation. *Proc Natl Acad Sci U S A* 109(3)**,** 823-828. doi: 10.1073/pnas.1118744109.

Ishitani, T., Kishida, S., Hyodo-Miura, J., Ueno, N., Yasuda, J., Waterman, M., et al. (2003). The TAK1-NLK mitogen-activated protein kinase cascade functions in the Wnt-5a/Ca(2+) pathway to antagonize Wnt/beta-catenin signaling. *Mol Cell Biol* 23(1)**,** 131-139.

Jakel, S., Albig, W., Kutay, U., Bischoff, F.R., Schwamborn, K., Doenecke, D., et al. (1999). The importin beta/importin 7 heterodimer is a functional nuclear import receptor for histone H1. *EMBO J* 18(9)**,** 2411-2423. doi: 10.1093/emboj/18.9.2411.

Jakel, S., and Gorlich, D. (1998). Importin beta, transportin, RanBP5 and RanBP7 mediate nuclear import of ribosomal proteins in mammalian cells. *EMBO J* 17(15)**,** 4491-4502. doi: 10.1093/emboj/17.15.4491.

Janer, A., Antonicka, H., Lalonde, E., Nishimura, T., Sasarman, F., Brown, G.K., et al. (2012). An RMND1 Mutation causes encephalopathy associated with multiple oxidative phosphorylation complex deficiencies and a mitochondrial translation defect. *Am J Hum Genet* 91(4)**,** 737-743. doi: 10.1016/j.ajhg.2012.08.020.

Jang, S.W., Yang, S.J., Ehlen, A., Dong, S., Khoury, H., Chen, J., et al. (2008). Serine/arginine protein-specific kinase 2 promotes leukemia cell proliferation by phosphorylating acinus and regulating cyclin A1. *Cancer Res* 68(12)**,** 4559-4570. doi: 10.1158/0008-5472.CAN-08-0021.

Jian, H., Liu, B., and Zhang, J. (2014a). Hypoxia and hypoxia-inducible factor 1 repress SEMA4B expression to promote non-small cell lung cancer invasion. *Tumour Biol* 35(5)**,** 4949-4955. doi: 10.1007/s13277-014-1651-4.

Jian, H., Zhao, Y., Liu, B., and Lu, S. (2014b). SEMA4b inhibits MMP9 to prevent metastasis of non-small cell lung cancer. *Tumour Biol* 35(11)**,** 11051-11056. doi: 10.1007/s13277-014-2409-8.

Keniry, A., Oxley, D., Monnier, P., Kyba, M., Dandolo, L., Smits, G., et al. (2012). The H19 lincRNA is a developmental reservoir of miR-675 that suppresses growth and Igf1r. *Nat Cell Biol* 14(7)**,** 659-665. doi: 10.1038/ncb2521.

Khrebtukova, I., Kuklin, A., Woychik, R.P., and Michaud, E.J. (1999). Alternative processing of the human and mouse raly genes(1). *Biochim Biophys Acta* 1447(1)**,** 107-112.

Klein, R.S., Rubin, J.B., Gibson, H.D., DeHaan, E.N., Alvarez-Hernandez, X., Segal, R.A., et al. (2001). SDF-1 alpha induces chemotaxis and enhances Sonic hedgehog-induced proliferation of cerebellar granule cells. *Development* 128(11)**,** 1971-1981.

Kloet, S.L., Whiting, J.L., Gafken, P., Ranish, J., and Wang, E.H. (2012). Phosphorylation-dependent regulation of cyclin D1 and cyclin A gene transcription by TFIID subunits TAF1 and TAF7. *Mol Cell Biol* 32(16)**,** 3358-3369. doi: 10.1128/MCB.00416-12.

Kool, M., Koster, J., Bunt, J., Hasselt, N.E., Lakeman, A., van Sluis, P., et al. (2008). Integrated genomics identifies five medulloblastoma subtypes with distinct genetic profiles, pathway signatures and clinicopathological features. *PLoS One* 3(8)**,** e3088. doi: 10.1371/journal.pone.0003088.

Kular, R.K., Cvetanovic, M., Siferd, S., Kini, A.R., and Opal, P. (2009). Neuronal differentiation is regulated by leucine-rich acidic nuclear protein (LANP), a member of the inhibitor of histone acetyltransferase complex. *J Biol Chem* 284(12)**,** 7783-7792. doi: 10.1074/jbc.M806150200.

Lapik, Y.R., Misra, J.M., Lau, L.F., and Pestov, D.G. (2007). Restricting conformational flexibility of the switch II region creates a dominant-inhibitory phenotype in Obg GTPase Nog1. *Mol Cell Biol* 27(21)**,** 7735-7744. doi: 10.1128/MCB.01161-07.

LaRochelle, W.J., Jeffers, M., Corvalan, J.R., Jia, X.C., Feng, X., Vanegas, S., et al. (2002). Platelet-derived growth factor D: tumorigenicity in mice and dysregulated expression in human cancer. *Cancer Res* 62(9)**,** 2468-2473.

Le Hir, H., Izaurralde, E., Maquat, L.E., and Moore, M.J. (2000). The spliceosome deposits multiple proteins 20-24 nucleotides upstream of mRNA exon-exon junctions. *EMBO J* 19(24)**,** 6860-6869. doi: 10.1093/emboj/19.24.6860.

Lee, D.K., Nguyen, T., Lynch, K.R., Cheng, R., Vanti, W.B., Arkhitko, O., et al. (2001). Discovery and mapping of ten novel G protein-coupled receptor genes. *Gene* 275(1)**,** 83-91.

Lin, S., and Gregory, R.I. (2014). Methyltransferases modulate RNA stability in embryonic stem cells. *Nat Cell Biol* 16(2)**,** 129-131. doi: 10.1038/ncb2914.

Liu, J., Yue, Y., Han, D., Wang, X., Fu, Y., Zhang, L., et al. (2014). A METTL3-METTL14 complex mediates mammalian nuclear RNA N6-adenosine methylation. *Nat Chem Biol* 10(2)**,** 93-95. doi: 10.1038/nchembio.1432.

Maerki, S., Olma, M.H., Staubli, T., Steigemann, P., Gerlich, D.W., Quadroni, M., et al. (2009). The Cul3-KLHL21 E3 ubiquitin ligase targets aurora B to midzone microtubules in anaphase and is required for cytokinesis. *J Cell Biol* 187(6)**,** 791-800. doi: 10.1083/jcb.200906117.

Mahoney, W.M., Jr., Gunaje, J., Daum, G., Dong, X.R., and Majesky, M.W. (2013). Regulator of G-protein signaling - 5 (RGS5) is a novel repressor of hedgehog signaling. *PLoS One* 8(4)**,** e61421. doi: 10.1371/journal.pone.0061421.

Maier, V., Jolicoeur, C., Rayburn, H., Takegahara, N., Kumanogoh, A., Kikutani, H., et al. (2011). Semaphorin 4C and 4G are ligands of Plexin-B2 required in cerebellar development. *Mol Cell Neurosci* 46(2)**,** 419-431. doi: 10.1016/j.mcn.2010.11.005.

Manoranjan, B., Venugopal, C., McFarlane, N., Doble, B.W., Dunn, S.E., Scheinemann, K., et al. (2012). Medulloblastoma stem cells: where development and cancer cross pathways. *Pediatr Res* 71(4 Pt 2)**,** 516-522. doi: 10.1038/pr.2011.62.

Marzluff, W.F., Gongidi, P., Woods, K.R., Jin, J., and Maltais, L.J. (2002). The human and mouse replication-dependent histone genes. *Genomics* 80(5)**,** 487-498.

Mathew, R., Hartmuth, K., Mohlmann, S., Urlaub, H., Ficner, R., and Luhrmann, R. (2008). Phosphorylation of human PRP28 by SRPK2 is required for integration of the U4/U6-U5 tri-snRNP into the spliceosome. *Nat Struct Mol Biol* 15(5)**,** 435-443. doi: 10.1038/nsmb.1415.

Matouk, I.J., Raveh, E., Abu-lail, R., Mezan, S., Gilon, M., Gershtain, E., et al. (2014). Oncofetal H19 RNA promotes tumor metastasis. *Biochim Biophys Acta* 1843(7)**,** 1414-1426. doi: 10.1016/j.bbamcr.2014.03.023.

McIlwain, D.R., Pan, Q., Reilly, P.T., Elia, A.J., McCracken, S., Wakeham, A.C., et al. (2010). Smg1 is required for embryogenesis and regulates diverse genes via alternative splicing coupled to nonsense-mediated mRNA decay. *Proc Natl Acad Sci U S A* 107(27)**,** 12186-12191. doi: 10.1073/pnas.1007336107.

Menghi, F., Jacques, T.S., Barenco, M., Schwalbe, E.C., Clifford, S.C., Hubank, M., et al. (2011). Genome-wide analysis of alternative splicing in medulloblastoma identifies splicing patterns characteristic of normal cerebellar development. *Cancer Res* 71(6)**,** 2045-2055. doi: 10.1158/0008-5472.CAN-10-2519.

Miller, H.B., Robinson, T.J., Gordan, R., Hartemink, A.J., and Garcia-Blanco, M.A. (2011). Identification of Tat-SF1 cellular targets by exon array analysis reveals dual roles in transcription and splicing. *RNA* 17(4)**,** 665-674. doi: 10.1261/rna.2462011.

Mimeault, M., and Batra, S.K. (2011). Complex oncogenic signaling networks regulate brain tumor-initiating cells and their progenies: pivotal roles of wild-type EGFR, EGFRvIII mutant and hedgehog cascades and novel multitargeted therapies. *Brain Pathol* 21(5)**,** 479-500. doi: 10.1111/j.1750-3639.2011.00505.x.

Mourtada-Maarabouni, M., Keen, J., Clark, J., Cooper, C.S., and Williams, G.T. (2006). Candidate tumor suppressor LUCA-15/RBM5/H37 modulates expression of apoptosis and cell cycle genes. *Exp Cell Res* 312(10)**,** 1745-1752. doi: 10.1016/j.yexcr.2006.02.009.

Muhlhausser, P., Muller, E.C., Otto, A., and Kutay, U. (2001). Multiple pathways contribute to nuclear import of core histones. *EMBO Rep* 2(8)**,** 690-696. doi: 10.1093/embo-reports/kve168.

Muto, A., Aoki, Y., and Watanabe, S. (2007). Mouse Rab11-FIP4 regulates proliferation and differentiation of retinal progenitors in a Rab11-independent manner. *Dev Dyn* 236(1)**,** 214-225. doi: 10.1002/dvdy.21009.

Northcott, P.A., Korshunov, A., Witt, H., Hielscher, T., Eberhart, C.G., Mack, S., et al. (2011). Medulloblastoma comprises four distinct molecular variants. *J Clin Oncol* 29(11)**,** 1408-1414. doi: 10.1200/JCO.2009.27.4324.

Northcott, P.A., Shih, D.J., Remke, M., Cho, Y.J., Kool, M., Hawkins, C., et al. (2012). Rapid, reliable, and reproducible molecular sub-grouping of clinical medulloblastoma samples. *Acta Neuropathol* 123(4)**,** 615-626. doi: 10.1007/s00401-011-0899-7.

Ortega, A., Niksic, M., Bachi, A., Wilm, M., Sanchez, L., Hastie, N., et al. (2003). Biochemical function of female-lethal (2)D/Wilms' tumor suppressor-1-associated proteins in alternative pre-mRNA splicing. *J Biol Chem* 278(5)**,** 3040-3047. doi: 10.1074/jbc.M210737200.

Ozawa, P.M., Ariza, C.B., Ishibashi, C.M., Fujita, T.C., Banin-Hirata, B.K., Oda, J.M., et al. (2014). Role of CXCL12 and CXCR4 in normal cerebellar development and medulloblastoma. *Int J Cancer*. doi: 10.1002/ijc.29333.

Parisi, C., Giorgi, C., Batassa, E.M., Braccini, L., Maresca, G., D'Agnano, I., et al. (2011). Ago1 and Ago2 differentially affect cell proliferation, motility and apoptosis when overexpressed in SH-SY5Y neuroblastoma cells. *FEBS Lett* 585(19)**,** 2965-2971. doi: 10.1016/j.febslet.2011.08.003.

Park, J.Y., Lee, J.E., Park, J.B., Yoo, H., Lee, S.H., and Kim, J.H. (2014). Roles of Long Non-Coding RNAs on Tumorigenesis and Glioma Development. *Brain Tumor Res Treat* 2(1)**,** 1-6. doi: 10.14791/btrt.2014.2.1.1.

Ping, X.L., Sun, B.F., Wang, L., Xiao, W., Yang, X., Wang, W.J., et al. (2014). Mammalian WTAP is a regulatory subunit of the RNA N6-methyladenosine methyltransferase. *Cell Res* 24(2)**,** 177-189. doi: 10.1038/cr.2014.3.

Plass, C., Pfister, S.M., Lindroth, A.M., Bogatyrova, O., Claus, R., and Lichter, P. (2013). Mutations in regulators of the epigenome and their connections to global chromatin patterns in cancer. *Nat Rev Genet* 14(11)**,** 765-780. doi: 10.1038/nrg3554.

Privette Vinnedge, L.M., Kappes, F., Nassar, N., and Wells, S.I. (2013). Stacking the DEK: from chromatin topology to cancer stem cells. *Cell Cycle* 12(1)**,** 51-66. doi: 10.4161/cc.23121.

Raciti, G.A., Bera, T.K., Gavrilova, O., and Pastan, I. (2011). Partial inactivation of Ankrd26 causes diabetes with enhanced insulin responsiveness of adipose tissue in mice. *Diabetologia* 54(11)**,** 2911-2922. doi: 10.1007/s00125-011-2263-9.

Reuven, N., Adler, J., Meltser, V., and Shaul, Y. (2013). The Hippo pathway kinase Lats2 prevents DNA damage-induced apoptosis through inhibition of the tyrosine kinase c-Abl. *Cell Death Differ* 20(10)**,** 1330-1340. doi: 10.1038/cdd.2013.83.

Roussel, M.F., and Hatten, M.E. (2011). Cerebellum development and medulloblastoma. *Curr Top Dev Biol* 94**,** 235-282. doi: 10.1016/B978-0-12-380916-2.00008-5.

Saha, A.K., Kappes, F., Mundade, A., Deutzmann, A., Rosmarin, D.M., Legendre, M., et al. (2013). Intercellular trafficking of the nuclear oncoprotein DEK. *Proc Natl Acad Sci U S A* 110(17)**,** 6847-6852. doi: 10.1073/pnas.1220751110.

Sengupta, R., Dubuc, A., Ward, S., Yang, L., Northcott, P., Woerner, B.M., et al. (2012). CXCR4 activation defines a new subgroup of Sonic hedgehog-driven medulloblastoma. *Cancer Res* 72(1)**,** 122-132. doi: 10.1158/0008-5472.CAN-11-1701.

Seo, S.B., McNamara, P., Heo, S., Turner, A., Lane, W.S., and Chakravarti, D. (2001). Regulation of histone acetylation and transcription by INHAT, a human cellular complex containing the set oncoprotein. *Cell* 104(1)**,** 119-130.

Singer, J.D., Gurian-West, M., Clurman, B., and Roberts, J.M. (1999). Cullin-3 targets cyclin E for ubiquitination and controls S phase in mammalian cells. *Genes Dev* 13(18)**,** 2375-2387.

Sumara, I., Quadroni, M., Frei, C., Olma, M.H., Sumara, G., Ricci, R., et al. (2007). A Cul3-based E3 ligase removes Aurora B from mitotic chromosomes, regulating mitotic progression and completion of cytokinesis in human cells. *Dev Cell* 12(6)**,** 887-900. doi: 10.1016/j.devcel.2007.03.019.

Takanashi, M., Oikawa, K., Fujita, K., Kudo, M., Kinoshita, M., and Kuroda, M. (2009). Heterochromatin protein 1gamma epigenetically regulates cell differentiation and exhibits potential as a therapeutic target for various types of cancers. *Am J Pathol* 174(1)**,** 309-316. doi: 10.2353/ajpath.2009.080148.

Tanikawa, C., Espinosa, M., Suzuki, A., Masuda, K., Yamamoto, K., Tsuchiya, E., et al. (2012). Regulation of histone modification and chromatin structure by the p53–PADI4 pathway. *Nat Commun* 3**,** 676. doi: 10.1038/ncomms1676.

Taylor, M., Northcott, P., Korshunov, A., Remke, M., Cho, Y.-J., and Clifford, S. (2012). Molecular subgroups of medulloblastoma: the current consensus. *Acta Neuropathol* 123**,** 465 - 472.

Tenzer, S., Moro, A., Kuharev, J., Francis, A.C., Vidalino, L., Provenzani, A., et al. (2013). Proteome-wide characterization of the RNA-binding protein RALY-interactome using the in vivo-biotinylation-pulldown-quant (iBioPQ) approach. *J Proteome Res* 12(6)**,** 2869-2884. doi: 10.1021/pr400193j.

Varelas, X., Samavarchi-Tehrani, P., Narimatsu, M., Weiss, A., Cockburn, K., Larsen, B.G., et al. (2010). The Crumbs complex couples cell density sensing to Hippo-dependent control of the TGF-beta-SMAD pathway. *Dev Cell* 19(6)**,** 831-844. doi: 10.1016/j.devcel.2010.11.012.

Wang, C., Pan, Y., and Wang, B. (2010). Suppressor of fused and Spop regulate the stability, processing and function of Gli2 and Gli3 full-length activators but not their repressors. *Development* 137(12)**,** 2001-2009. doi: 10.1242/dev.052126.

Wang, Y., Li, P., Wang, S., Hu, J., Chen, X.A., Wu, J., et al. (2012). Anticancer peptidylarginine deiminase (PAD) inhibitors regulate the autophagy flux and the mammalian target of rapamycin complex 1 activity. *J Biol Chem* 287(31)**,** 25941-25953. doi: 10.1074/jbc.M112.375725.

Wang, Y., Li, Y., Toth, J.I., Petroski, M.D., Zhang, Z., and Zhao, J.C. (2014). N6-methyladenosine modification destabilizes developmental regulators in embryonic stem cells. *Nat Cell Biol* 16(2)**,** 191-198. doi: 10.1038/ncb2902.

Wang, Y., Wysocka, J., Sayegh, J., Lee, Y.H., Perlin, J.R., Leonelli, L., et al. (2004). Human PAD4 regulates histone arginine methylation levels via demethylimination. *Science* 306(5694)**,** 279-283. doi: 10.1126/science.1101400.

Xiong, H.Y., Alipanahi, B., Lee, L.J., Bretschneider, H., Merico, D., Yuen, R.K., et al. (2015). RNA splicing. The human splicing code reveals new insights into the genetic determinants of disease. *Science* 347(6218)**,** 1254806. doi: 10.1126/science.1254806.

Yang, H., Sasaki, T., Minoshima, S., and Shimizu, N. (2007). Identification of three novel proteins (SGSM1, 2, 3) which modulate small G protein (RAP and RAB)-mediated signaling pathway. *Genomics* 90(2)**,** 249-260. doi: 10.1016/j.ygeno.2007.03.013.

Yoshikawa, H., Komatsu, W., Hayano, T., Miura, Y., Homma, K., Izumikawa, K., et al. (2011). Splicing factor 2-associated protein p32 participates in ribosome biogenesis by regulating the binding of Nop52 and fibrillarin to preribosome particles. *Mol Cell Proteomics* 10(8)**,** M110 006148. doi: 10.1074/mcp.M110.006148.

Yuan, L., Zhang, H., Liu, J., Rubin, J.B., Cho, Y.J., Shu, H.K., et al. (2013). Growth factor receptor-Src-mediated suppression of GRK6 dysregulates CXCR4 signaling and promotes medulloblastoma migration. *Mol Cancer* 12**,** 18. doi: 10.1186/1476-4598-12-18.

Zhang, A., Yeung, P.L., Li, C.W., Tsai, S.C., Dinh, G.K., Wu, X., et al. (2004). Identification of a novel family of ankyrin repeats containing cofactors for p160 nuclear receptor coactivators. *J Biol Chem* 279(32)**,** 33799-33805. doi: 10.1074/jbc.M403997200.

Zhang, Y., Woodford, N., Xia, X., and Hamburger, A.W. (2003). Repression of E2F1-mediated transcription by the ErbB3 binding protein Ebp1 involves histone deacetylases. *Nucleic Acids Res* 31(8)**,** 2168-2177.

Supplementary Table 1 Deregulated genes/entities belonging to the set A. Information about Probe ID, Gene Symbol and Fold-Change (FC) are shown in this table for each one of the deregulated gene belonging to the Set A.

ProbeID	GeneSymbol	FC ([Ptch1 +- Tis21 --] vs [Ptch1 +- Tis21 ++])
A_52_P404363	Cdc42bpb	-4.3387275
A_51_P176448	AK038757	-12.789862
A_52_P618774	Hs1bp3	-22.129265
A_52_P136782	Rgs5	2.257812
A_51_P286215	Zfyve20	6.577763
A_51_P266155	Fat4	-18.18666
A_51_P383014	Arhgap26	-42.585026
A_51_P302308	9630033F20Rik (Tigar)	-25.21376
A_51_P199987	Gucy1a3	3.4854343
A_51_P109496	Gm13139	-5.744404
A_51_P286826	March10	-8.464081
A_52_P708817	Frk	-41.566597
A_51_P462516	Senp7	2.3042748
A_51_P130459	Vdac1	1.9172508
A_52_P494841	NAP030047-1	-9.371233
A_51_P296274	Sgsm2	10.392676
A_52_P83959	Taf7	19.883877
A_51_P403564	Lhx5	1.4888201
A_52_P670725	Gtpbp4	3.0447798
A_52_P1123475	AK047771	10.073194
A_52_P593337	Lyrm7	-29.024801
A_52_P53144	Gcnt3	-26.793251
A_52_P551851	Tspan11	28.112204
A_51_P144330	4930431F10Rik	-23.481102
A_52_P566005	Taok2	1.8026309
A_51_P490070	Xlr3c	3.7286766
A_51_P441469	Jmy	-29.81633
A_51_P464588	Dnajc28	11.893069
A_52_P360921	Rbm5	2.6342618
A_52_P201106	Ccdc171	3.8724778
A_52_P664404	Zfp286	1.7244819
A_51_P385059	Zfhx2as	-7.3103805
A_51_P216147	Emd	1.9309781

A_51_P208145	Pmel	-52.71158
A_52_P51896	AK082682	-24.289986
A_52_P157158	Dsc2	-17.820856
A_51_P181205	9130401M01Rik	2.6138315
A_51_P464118	5830472F04Rik	7.881003
A_51_P420085	Sltm	2.26791
A_52_P477709	Dek	2.0809693
A_51_P210143	Syne2	3.9390473
A_52_P346069	Zpbp2	-50.937298
A_52_P166275	Gpr82	-13.744616
A_52_P755756	AK087246	-11.792826
A_51_P329869	Dgkq	5.127107
A_52_P61758	Kctd5	9.1257515
A_51_P338664	Hist3h2ba	2.9225478
A_52_P87713	Timp1	-7.515524
A_51_P499755	Anp32a	4.9533424
A_51_P104727	Ehbp1	2.0839832
A_51_P105339	Tomm22	2.647325
A_51_P461201	Cdc27	2.0909421
A_52_P235108	Vps35	2.063168
A_52_P675052	Golgb1	7.87962
A_51_P142196	H19	-30.557398
A_51_P470589	Lars	3.0106843
A_52_P169869	Ube2t	-80.0219
A_51_P255875	Padi4	-25.649277
A_52_P582705	Serbp1	-31.619617
A_52_P339996	Slc6a6	2.8363895
A_51_P291388	Pafah1b1	2.7477632
A_52_P619738	Gramd3	2.7288601
A_52_P663303	Wdr60	2.2419453
A_52_P97489	Eif2c1	3.4095387
A_52_P5855	Egfr	-8.421739
A_51_P348749	Llgl2	-15.929397
A_51_P141580	Dpp10	2.6221642
A_52_P434073	Lnx1	-13.800558
A_52_P661503	Deptor	4.7166576
A_52_P50496	H2-K1	-3.9359083
A_52_P1115594	AK047731	-15.971685
A_52_P97595	Egflam	-3.6955972
A_51_P452352	Rrp1	2.7214644
A_51_P368660	Mphosph10	2.0928893
A_52_P400355	3110035E14Rik	2.4135242

A_52_P70231	Adamts5	-54.697617
A_51_P246060	Dazl	-10.899264
A_51_P115268	Ankrd26	2.2836397
A_51_P214612	AK032608	2.5137906
A_52_P398334	Ccdc157	-8.405986
A_52_P75127	Tcp11	-95.24133
A_52_P151278	Lrch4	-19.647413
A_52_P632191	Nfx1	-2.3208349
A_52_P399646	Rmnd1	2.5840914
A_52_P1034794	5430434G16Rik	3.0165286
A_51_P226417	Rraga	1.8614218
A_52_P609448	Brwd1	2.179845
A_52_P299888	Pag1	-13.040045
A_52_P86384	Nr2c2	3.3261437
A_52_P43661	Ncor1	-30.37849
A_51_P229280	Eif3a	2.0501459
A_52_P306305	Akap2	2.4785964
A_51_P502993	Ankrd24	2.2780094
A_52_P23177	Acaca	-32.10315
A_52_P1020153	AK050110	-34.570435
A_51_P215496	Rab18	1.7599123
A_51_P229957	Olfr541	-11.346127
A_52_P576886	Smurf2	2.5268075
A_52_P524345	Olfr670	-14.145674
A_52_P232813	Cxcl3	-4.038158
A_52_P499821	Erg	2.045569
A_52_P447196	Col4a6	-3.2609994
A_52_P466171	Ints6	-29.568296
A_51_P144712	Gbf1	2.9894059
A_52_P490470	NP614311	-100.73444
A_51_P354382	Csda	6.5801787
A_51_P326191	Serpina3g	-10.784577
A_51_P410205	Hsd11b2	4.629768
A_51_P392593	Dmxl1	-31.071264
A_52_P9347	Ddx23	3.567187
A_51_P443387	Wtap	-2.2928643
A_52_P410859	B3gntl1	-5.5193987
A_52_P524366	TC1698027	49.320824
A_51_P325856	1810033B17Rik (Mcemp1)	-10.32224
A_51_P123494	Ttll5	-35.24531
A_52_P618947	Olfr1487	31.364109

A_52_P94149	4933415F23Rik	-16.782778
A_52_P260126	U2surp	2.4091258
A_52_P505907	Gigyf2	3.8953524
A_51_P132530	Cldn22	-52.257294
A_51_P347452	Htatsf1	2.2065017
A_52_P431116	Col23a1	-24.499304
A_51_P234386	AK052113	-13.513184
A_51_P184573	Ube2o	2.7823486
A_52_P121525	Strbp	1.9385884
A_51_P491987	Ripk3	7.0459323
A_51_P510437	Slc25a15	-15.014685
A_52_P40832	Rab11fip4	-2.0099378
A_52_P648688	Zc3h12d	-8.824186
A_51_P392701	AK036490	-3.9222114
A_52_P1067724	AK034311	11.082592
A_51_P337246	Raly	3.0295527
A_52_P820923	1700001L05Rik	-5.605956
A_52_P31543	Btg2	-14.55926
A_52_P1122623	2010013B24Rik	-5.3051004
A_51_P361620	Hist2h2bb	-11.467025
A_51_P230496	Pth	-21.315046
A_51_P291139	Upf3b	2.233692
A_52_P397231	Cbx3	-14.688863
A_51_P196243	Ckap5	3.209575
A_51_P296456	Ankrd11	2.7607532
A_51_P161225	Ddx46	3.677435
A_52_P335892	Luc7l2	2.7024581
A_52_P138046	Ppp1r13l	-9.435442
A_51_P443782	4921523P09Rik	-33.79208
A_51_P341688	Tiaf2	6.8558946
A_51_P262630	Ceacam3	13.851112
A_51_P306247	Nrcam	3.6596556
A_52_P671784	Adamts10	-3.0174506
A_51_P343356	Isoc2b	1.6532197
A_52_P177988	Fam179b	2.6087596
A_52_P104155	Mettl14	-14.64095
A_52_P256426	Gm9182	-16.936632
A_51_P384718	Efna4	-11.366827
A_52_P335587	Prrx1	-19.576757
A_52_P18807	Eif3c	2.06448
A_52_P425064	Lats2	-21.153925
A_52_P795474	AK052970	4.083203

A_51_P119016	Usp36	2.4442112
A_52_P654720	1500004A13Rik (Syt11)	-8.627816
A_51_P321512	Rab11fip2	-41.566597
A_52_P575296	Sik2	-17.441523
A_51_P299954	Tcl1b3	-8.998422
A_52_P466641	Smg1	-11.584062
A_52_P415440	Rps12	8.925112
A_51_P437978	Agtr2	28.945824
A_52_P292853	Napepld	-17.700521
A_51_P365440	Sema4b	-12.363551
A_52_P515497	Atp1a1	1.6794865
A_51_P192694	AK084634	-19.387697
A_51_P384584	Med29	-8.233592
A_52_P279068	Gpatch2	2.769899
A_52_P365925	TC1686295	5.847777
A_52_P360724	NAP029213-1	-36.78141
A_51_P295858	Unknown	-8.901256
A_52_P636948	Pdgfd	15.714823
A_51_P457244	Xlr4b	3.2490456
A_51_P162718	Bsn	-9.046874
A_52_P367147	A930013B10Rik	-9.895246
A_52_P470373	Nlk	2.3255427
A_51_P451301	Fam168a	3.1332333
A_51_P129999	Sh2d7	-7.5248685
A_52_P3214	Ipo7	3.5059268
A_52_P456898	Lactb	2.88868
A_51_P248865	Foxf2	1.8928239
A_51_P119923	Pa2g4	2.5200555
A_52_P602771	Srpk2	1.8982989
A_51_P172502	Cxcl12	1.8274761
